# Adiposity in mares induces insulin dysregulation and mitochondrial dysfunction which can be mitigated by nutritional intervention

**DOI:** 10.1038/s41598-024-64628-x

**Published:** 2024-06-18

**Authors:** Kyle Fresa, Giovana D. Catandi, Luke Whitcomb, Raul A. Gonzalez-Castro, Adam J. Chicco, Elaine M. Carnevale

**Affiliations:** Department of Biomedical Sciences, Colorado State University, Fort Collins, CO 80523 USA

**Keywords:** Equine, Muscle, Systemic, Metabolism, Insulin dysregulation, Obesity, Mitochondrial dysfunction, Obesity, Metabolic disorders, Nutrition disorders, Nutrition

## Abstract

Obesity is a complex disease associated with augmented risk of metabolic disorder development and cellular dysfunction in various species. The goal of the present study was to investigate the impacts of obesity on the metabolic health of old mares as well as test the ability of diet supplementation with either a complex blend of nutrients designed to improve equine metabolism and gastrointestinal health or L-carnitine alone to mitigate negative effects of obesity. Mares (n = 19, 17.9 ± 3.7 years) were placed into one of three group: normal-weight (NW, n = 6), obese (OB, n = 7) or obese fed a complex diet supplement for 12 weeks (OBD, n = 6). After 12 weeks and completion of sample collections, OB mares received L-carnitine alone for an additional 6 weeks. Obesity in mares was significantly associated with insulin dysregulation, reduced muscle mitochondrial function, and decreased skeletal muscle oxidative capacity with greater ROS production when compared to NW. Obese mares fed the complex diet supplement had better insulin sensivity, greater cell lipid metabolism, and higher muscle oxidative capacity with reduced ROS production than OB. L-carnitine supplementation alone did not significantly alter insulin signaling, but improved lipid metabolism and muscle oxidative capacity with reduced ROS. In conclusion, obesity is associated with insulin dysregulation and altered skeletal muscle metabolism in older mares. However, dietary interventions are an effective strategy to improve metabolic status and skeletal muscle mitochondrial function in older mares.

## Introduction

Obesity, or excess adiposity, is associated with health concerns in various species due to metabolic and cellular functional disorders. In horses, obesity affects up to 72% of certain populations in the United States and Europe, although percentages vary substantially among populations and breeds^1,2^. Horses are monogastric mammals that develop similar clinical pathologies as humans regarding metabolic disorders and insulin signaling^3^. Obesity in horses is commonly associated with insulin dysregulation (ID), increasing risks for hypertension, dyslipidemia, and vascular disease^4^. Horses with metabolic disorders rarely exhibit type-2 diabetes mellitus as observed in humans^5^. However, other severe pathologies can occur concurrent with ID, such as equine metabolic syndrome and endocrinopathic laminitis, which exhibit similar vascular disturbances as observed in cardiovascular diseases in humans^6^. The health risks of obesity are most notable in the human population, as the incidence of obesity has increased in the past 50 years, and now affects approximately 42% of adults in the United States^7^. Human obesity is associated with a variety of pathologies, such as insulin resistance, cardiovascular disease, and hypertension, although individual risk is highly variable based on factors such as age, sex, genetics, and exercise volume^8–10^. Health outcomes associated with metabolic disorders differ among mammalian species and present inherent difficulties to study treatments to improve insulin sensitivity^11^. However, the metabolic responses of horses and humans to insulin dysfunction are potentially similar, especially when factors such as obesity, age, and sex are implicated.

Cellular metabolism is often altered with excess adiposity, displaying a shift toward fatty acid oxidation, impaired glucose metabolism, and/or reduced mitochondrial activity^12,13^. Potential underlying factors of disease associated with obesity involve disturbances in inflammatory and insulin signaling, which impair glucose homeostasis^14–16^. Skeletal muscle is rich in mitochondria for energy regulation, playing a crucial role in glucose homeostasis^17–19^. Skeletal muscle is the largest tissue in the body, representing approximately 40% of body mass in horses and humans, but the proportion of muscle is influenced by age, sex, fitness, genetics, and other factors^19,20^. Glucose uptake in mammalian skeletal muscle is regulated by insulin, which induces the translocation of glucose transporter type 4 (GLUT4) from intracellular compartments to the plasma membrane to support glucose influx^21^. Similar to humans, decreased insulin sensitivity in horses is associated with impaired GLUT4-mediated glucose uptake and increased reactive oxygen species (ROS) production in skeletal muscle, which can result in damage to DNA, proteins, and lipids^22,23^. An imbalance of ROS production and antioxidant response can drive oxidative stress, facilitating insulin dysregulation, mitochondrial dysfunction, and cell death^24^. The specific mechanisms that cause insulin dysregulation in horses are still an area of research. The availability of older animals with large muscle mass and similar metabolic dysfunction risks supports the use of older and obese horses as animal models to study systemic and cellular mechanisms associated with insulin sensivity.

Reducing energy intake and exercising are often considered essential components for treating obesity and insulin dysregulation^16,25,26^. Dietary supplements with specific compounds are potentially valuable for improving metabolic status. However, dietary components often have synergistic and complex interactions that are poorly understood in mammals. For instance, dietary chromium and L-carnitine supplementation appear to have positive effects on cell metabolism and mitochondrial function in obese mice and women^27–30^. L-carnitine facilitates the transport of long-chain fatty acids into mitochondria for energy production and is essential for fat metabolism in mammalian species^31^. L-carnitine also has antioxidant properties, as it scavenges free-radicals, reduces production of free-radicals in stressed mitochondria, and induces cellular production of antioxidant enzymes^32^. The impact of L-carnitine supplementation on metabolism and oxidative stress in skeletal muscle have not been described in the horse. In previous studies, we demonstrated positive effects on mitochondrial energy production and reduced oxidative stress in the ovarian follicular cells of older mares after supplementation with antioxidants, amino acids (including L-carnitine), omega-3-rich oils, probiotics, prebiotics, vitamins, and minerals^33^. These findings suggest that the impacts of other causes of metabolic stress, such as adiposity, could be mitigated using nutritional interventions.

We hypothesized that equine obesity would result in insulin dysregulation, altered lipid metabolism, and mitochondrial dyfunction, and that short-term supplementation of nutrients designed to improve equine metabolism and gastrointestinal health could mitigate these negative effects, independent of weight loss or exercise. We used a combination of morphometric and systemic assays, protein analysis, and high-resolution respirometry to evaluate mitochondrial and cellular function resulting from obesity and the potential of dietary supplemention of a complex dietary supplement or L-carnitine to improve insulin dysregulation and muscle mitochondrial function in mares.

## Results

### Mare morphometric measurements

**Figure 1:**
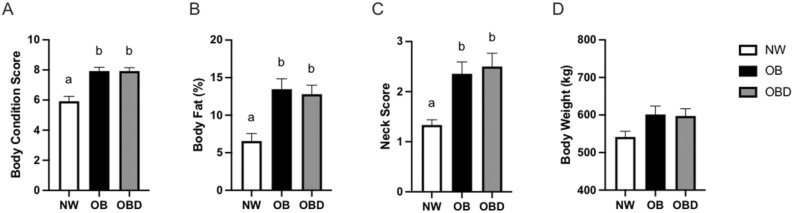
Morphometric and metabolic classification of mares. Morphometric measurements from normal-weight (NW), obese (OB) and obese diet supplemented (OBD) mares performed at week 12 of diet prior to sampling for (**a**) body condition score, (**b**) percentage of body fat calculated using a tailhead fat measurement (**c**), cresty neck score, (**d**) and body weight; ^ab^ superscripts indicate differences at *p* < 0.05. Bars represent mean ± SEM.

Mean age was similar among groups for normal-weight (NW), obese (OB), and obese diet supplemented (OBD) mares (17.8 ± 4.3, 18.1 ± 3.9, 17.7 ± 3.6 yr, respectively). Morphometric measurements were performed throughout the study to assess adiposity. Prior to sample collections, morphometric measurements of body condition score (BCS), percent body fat (BF), and neck score (NS) were higher (*p* ≤ 0.01) in obese groups when compared to NW (Fig. 1a–c). Body weight (BW) did not significantly differ (*p* = 0.1) due to varying mare sizes (Fig. 1d). Morphometric measurements were assessed every two weeks prior to sample collections to determine changes over time during feeding (Supplementary Figure S1). No differences were observed between OB and OBD for morphometric measurements, and a BCS of ≥ 7, characterizing obesity, was confirmed for all mares in OB and OBD before sample collections.

### Measurements of insulin dysregulation

**Figure 2:**
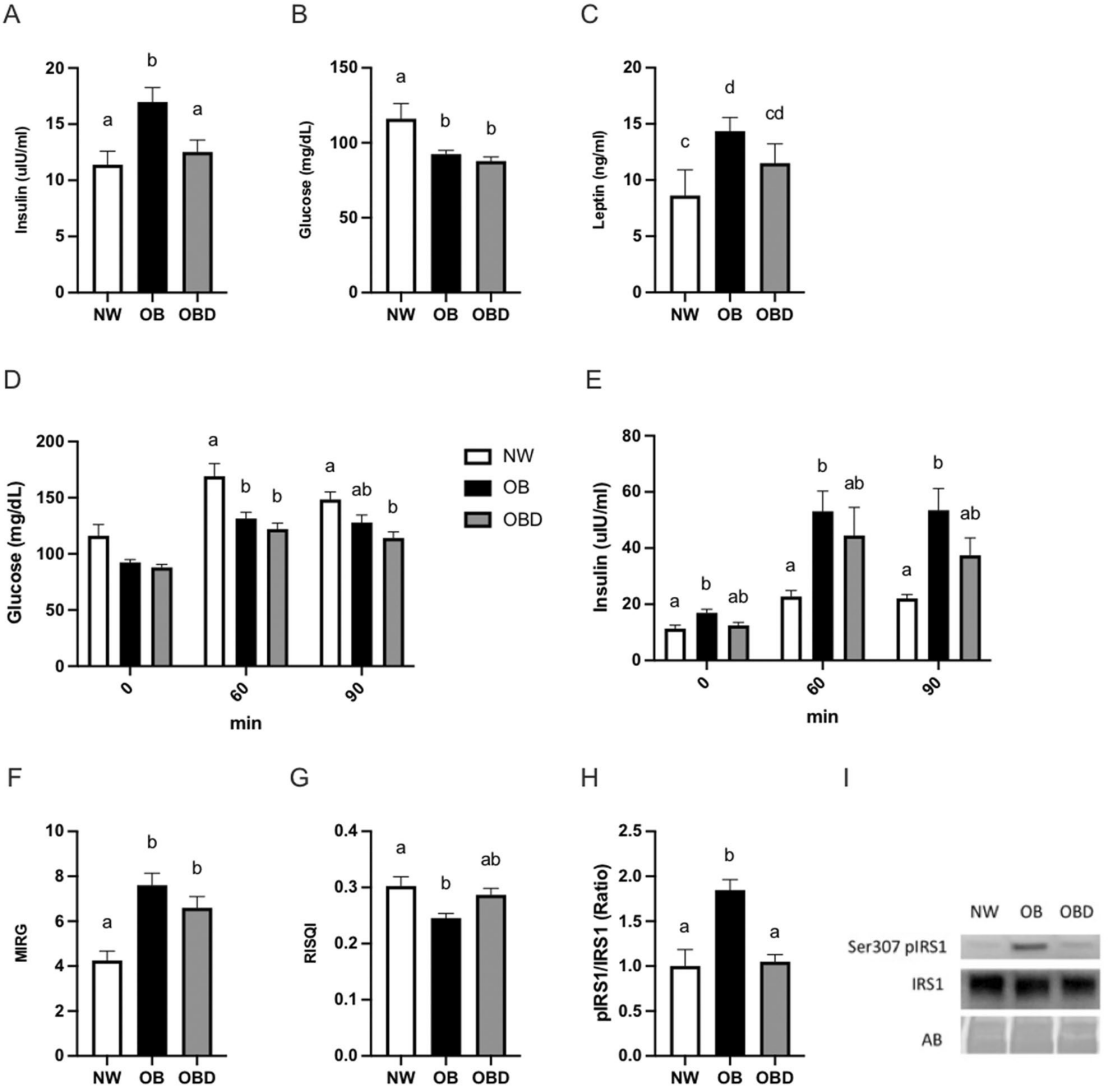
Metabolic hormone signaling and circulating metabolites. At 12 weeks of diet supplementation, normal-weight (NW), obese (OB) and obese diet supplemented (OBD) mares were fasted overnight before blood collection and analysis of basal concentrations of (**a**) insulin, (**b**) glucose, and (**c**) leptin; (**d**–**e**) glucose and insulin concentrations response within group after oral administration light corn syrup for an oral sugar test at 0 (basal), 60 and 90 min; Proxies, including (**f**) modified insulin to glucose ratio (MIRG) and (**g**) reciprocal of the square root of insulin (RISQI), used to quantify pancreatic response to glucose and insulin sensitivity, respectively; (**h**) ratio of phosphorylated IRS-1 to total IRS-1 in skeletal muscle; (**i**) Representative western blot for phosphorylation of IRS-1 to total IRS-1; ^ab^ Superscripts indicate differences at *p* < 0.05. ^cd^ Superscripts indicate differences at *p* = 0.08. Bars represent mean ± SEM.

The effect of adiposity and nutrient supplementation on insulin dysregulation and glucose response was assessed using multiple parameters. Systemic concentrations of insulin were measured at 12wk and were greater (*p* < 0.04) in OB than NW and OBD after overnight fasting (Fig. 2a). Fasting concentrations of glucose were greater (*p* < 0.03) in NW than OB and OBD (Fig. 2b). Circulating concentrations of leptin tended (*p* = 0.06) to be elevated in OB when compared to NW, but not OBD (*p* = 0.5, Fig. 2c). The insulin response to glucose was determined using an oral sugar test, based on measurements of glucose and insulin at 0, 60, and 90 min after administration of corn syrup (Fig. 2d,e)^34^. Insulin concentration above 45 μU/ml after 60 and 90 min was used as a threshold for insulin dysregulation^35^. No NW mares, six of seven OB mares (86%), and two of seven OBD mares (28%) were positive for insulin dysregulation (*p* = 0.005, Fig. 2e). Thus, OB were more frequently diagnosed with insulin dysregulation than NW mares, with a tendency (*p* = 0.09) for more OBD than OB to have an insulin response within normal limits after sugar consumption. The reciprocal of the square root of insulin (RISQI) proxy identified a reduction in insulin sensitivity in OB when compared to NW (*p* = 0.01) and a tendency (*p* = 0.06) for increased insulin sensitivity in OBD when compared to OB (Fig. 2g). Interestingly, the the modified insulin to glucose ratio (MIRG) proxy suggested increased pancreatic insulin release in both OB (*p* = 0.0005) and OBD (*p* = 0.01) when compared to NW (Fig. 2f). In skeletal muscle, expression of serine-307 phosphorylated insulin receptor substrate-1 (IRS1) relative to total IRS1 protein was similar between NW and OBD, and both were reduced (*p* < 0.002) when compared to OB (Fig. 2h,i).

### Systemic concentrations of fatty acids and carnitine species

**Figure 3:**
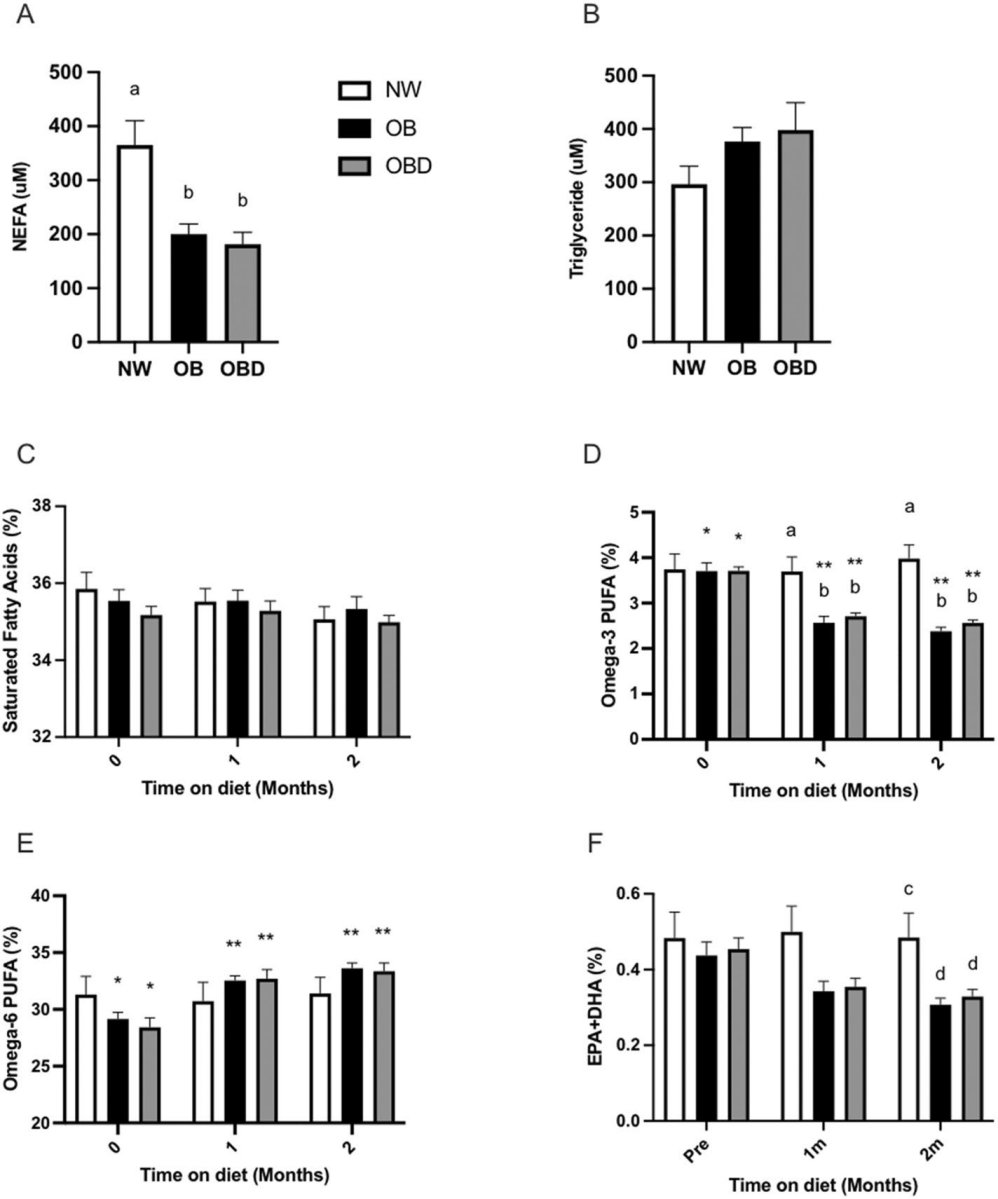
Fatty acid uptake and integration. Samples were collected after 12 weeks on diets for mares grouped into normal-weight (NW), obese (OB) and obese diet supplemented (OBD) after overnight fasting. Circulating (**a**) non-esterified fatty acids (NEFA) and (**b**) triglycerides were measured in plasma; Percentages of (**c**) saturated fatty acids, (**d**) omega-3 polyunsaturated fatty acids (PUFA), **(e)** omega-6 PUFA, and (**f**) EPA + DHA to total fatty acids were measured in red blood cell membranes. ^ab^ Superscripts indicate differences at *p* < 0.05 within time point. ^cd^ Superscripts indicate tendency at *p* < 0.09 within time points. *,** Asterisks indicate differences at *p* < 0.05 among months within individual groups. Bars represent mean ± SEM.

Fasting concentrations of circulating non-esterified fatty acids (NEFA) were lower (*p* < 0.004) in OB and OBD than NW (Fig. 3a). Plasma concentrations of triglycerides were not different (*p* = 0.2) among groups (Fig. 3b). Proportion of saturated fatty acids related to total fatty acids measured in red blood cells (RBC) were not different among groups and did not change over time (Fig. 3c). However, after 8 weeks of grain supplementation, the percentage of omega-3 polyunsaturated fatty acids (PUFA) was lower in OB and OBD when compared to the week 0 in RBC (*p* < 0.0001, Fig. 3d). In contrast, percentage of omega-6 PUFAs to total fatty acids increased in both OB (*p* = 0.001) and OBD (*p* < 0.0001) when compared to initial measurements in RBC (Fig. 3e). Proportional composition of eicosapentaenoic acid (EPA) + docosahexaenoic acid (DHA) in RBC tended (*p* = 0.06) to decrease in OB and OBD groups when compared to previous measurements (Fig. 3f). At 8 weeks, percentages of omega-3 PUFAs were higher in OB (*p* = 0.005) and OBD (*p* = 0.01) than NW (Fig. 3e) in RBC. However, no differences were observed among groups for percentage of omega-6 PUFAs. Percentage of EPA + DHA tended (*p* = 0.09) to decrease in OB and OBD groups when compared to NW at 8 weeks of diet intervention (Fig. 3f).

**Figure 4:**
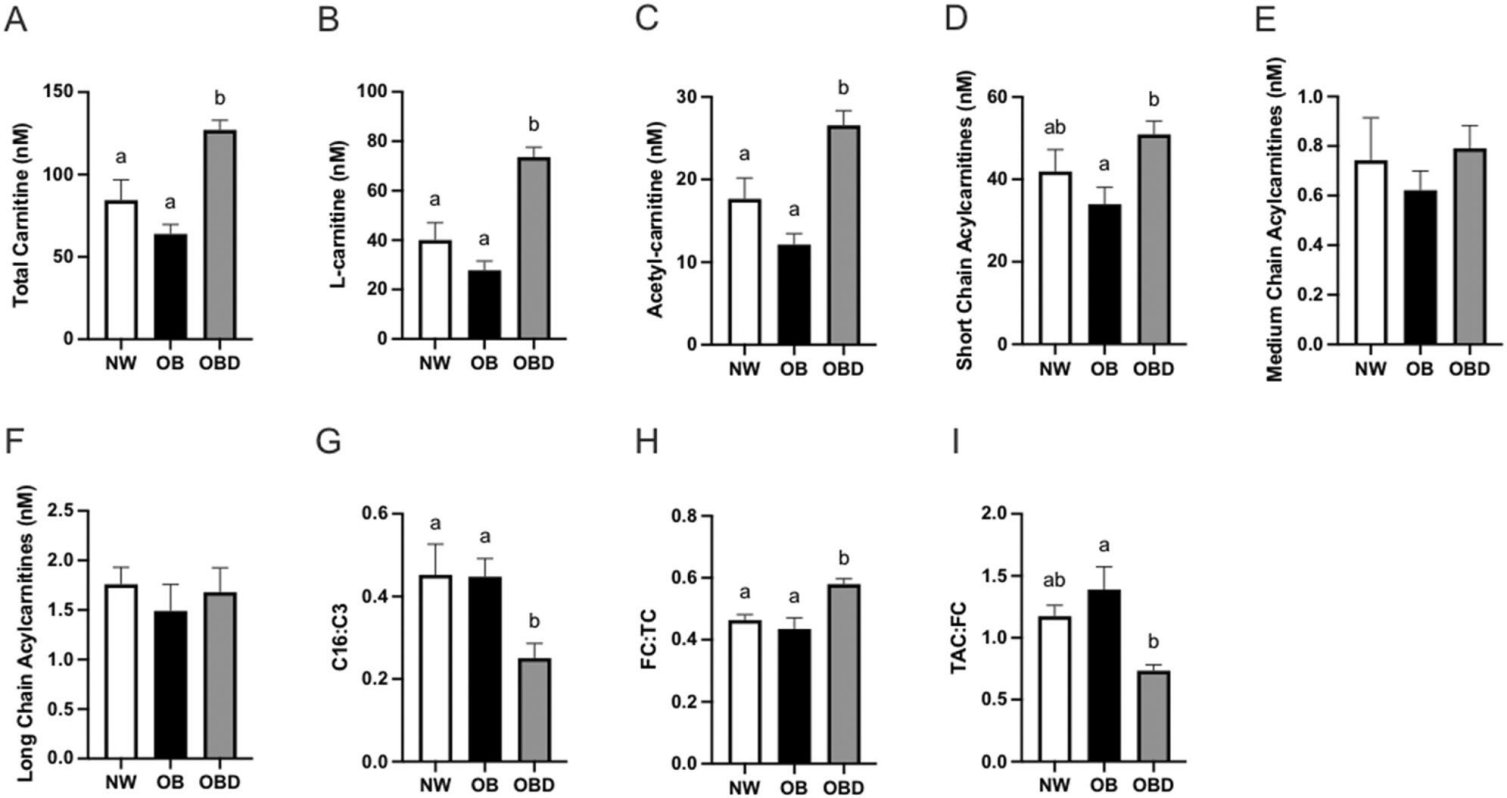
Acylcarnitine profiles. Serum acylcarnitine profiles were assessed 12 weeks after mares were placed on diets to maintain normal body weight (NW), obesity (OB) or obese diet supplemented (OBD). Serum samples were incubated and processed with an internal standard of known concentrations of acylcarnitines and analyzed using liquid chromatography mass spectrometry to determine concentrations of (**a**) total carnitine, (**b**) L-carnitine, (**c**) total acetyl carnitines, (**d**) short chain acylcarnitines, (**e**) medium chain acylcarnitines, and (**f**) long chain acylcarnitines; ratio of (**g**) C16:C3 which approximates completeness of β-oxidation, with lower values representing increased lipid metabolism efficiency; and ratios of (**h**) free carnitine to total carnitines (FC:TC) or (**i**) or total acetyl carnitines to free carnitine (TAC:FC) which represent carnitine availability. ^ab^ Superscripts indicate differences at *p* < 0.05. Bars represent mean ± SEM.

Circulating carnitine species were compared among groups to evaluate lipid metabolism at 12 weeks of dietary regime. Total carnitine species were increased (*p* ≤ 0.005) in OBD mares when compared to NW and OB (Fig. 4a). Circulating L-carnitine was higher (*p* < 0.0005) in OBD than NW and OB, demonstrating uptake from the dietary supplement (Fig. 4b). This was accompanied by higher concentrations of acetyl-carnitine in OBD relative to OB (*p* < 0.0001) and NW (*p* = 0.01, Fig. 4c). Differences in short chain acylcarnitine species were observed for OB and OBD (*p* = 0.02), although medium and long chain species were not significantly different among the groups (Fig. 4d–f). Plasma concentrations of individual acylcarnitine species for groups are shown in Table 1.

**Table 1:** Concentrations (nM, mean ± SEM) of individual acylcarnitine species in plasma of normal-weight (NW, n = 6), obese (OB, n = 7) and obese diet supplemented (OBD, n = 6) mares after at least 6 weeks on the respective diets; ^ab^ Superscripts within the same row indicate difference at *p* < 0.05.

	NW	OB	OBD
Short-chain acylcarnitines			
Acetyl (C2)	17.66 ± 2.52^a^	12.13 ± 1.34^a^	26.56 ± 1.76^b^
Propionyl (C3)	1.07 ± 0.15^a^	0.76 ± 0.09^a^	1.93 ± 0.13^b^
Succinyl (C4-DC)	21.36 ± 3.06	18.99 ± 3.83	18.18 ± 2.03
Hydroxybutyryl (C4-OH)	0.50 ± 0.19	0.39 ± 0.15	0.85 ± 0.16
Butanoyl (C4)	0.88 ± 0.13^a^	1.38 ± 0.27^ab^	2.48 ± 0.41^b^
Hydroxyisovaleryl (C5-OH)	0.10 ± 0.05^ab^	0.08 ± 0.02^a^	0.25 ± 0.04^b^
Isovaleryl (C5)	0.36 ± 0.05^a^	0.24 ± 0.04^a^	0.66 ± 0.02^b^
Medium-chain acylcarnitines			
Adipyl (C6-DC)	0.53 ± 0.16	0.56 ± 0.09	0.61 ± 0.09
Hexanoyl (C6)	0.05 ± 0.01	0.04 ± 0.01	0.06 ± 0.004
Decanoyl (C10)	0.08 ± 0.01	0.05 ± 0.01	0.07 ± 0.01
Dodecanoyl (C12)	0.08 ± 0.01	0.05 ± 0.01	0.06 ± 0.005
Long-chain acylcarnitines			
Tetradecenoyl (C14:1)	0.26 ± 0.04^a^	0.10 ± 0.01^b^	0.13 ± 0.02^b^
Tetradecanoyl (C14)	0.09 ± 0.01	0.06 ± 0.01	0.07 ± 0.01
Hexadecenoyl (C16:1)	0.16 ± 0.03	0.14 ± 0.03	0.11 ± 0.02
Palmitoyl (C16)	0.45 ± 0.05	0.42 ± 0.09	0.47 ± 0.05
Linoleoyl (C18:2)	0.13 ± 0.03	0.11 ± 0.01	0.18 ± 0.04
Octadecenoyl (C18:1)	0.68 ± 0.08	0.67 ± 0.12	0.73 ± 0.11

Ratios of key acylcarnitine species revealed differences in availability of L-carnitine and β-oxidation among groups. Ratios of C16:C3 were used to approximate completeness of β-oxidation, with lower values representing increased lipid metabolism efficiency due to higher amounts of oxidized C3 products. This ratio was lower (*p* ≤ 0.04) in OBD than OB and NW, suggesting that the diet supplement fed to OBD improved mitochondrial lipid metabolism (Fig. 4g). Higher (*p* < 0.02) free carnitine in relation to total carnitine was observed in OBD when compared to OB and NW (Fig. 4h). The ratio of total acylcarnitine to free L-carnitine was lower (*p* = 0.004) in OBD when compared to OB (Fig. 4i), and tended (*p* = 0.06) to be lower between OB and NW. Overall, the addition of targeted supplementation combining vitamins, trace minerals, amino acids (including L-carnitine), antioxidants, omega-3 fatty acids, prebiotics and probiotics in OBD mares increased the availability of free L-carnitine and improved the efficiency of lipid metabolism.

### Mitochondrial function in skeletal muscle

**Figure 5:**
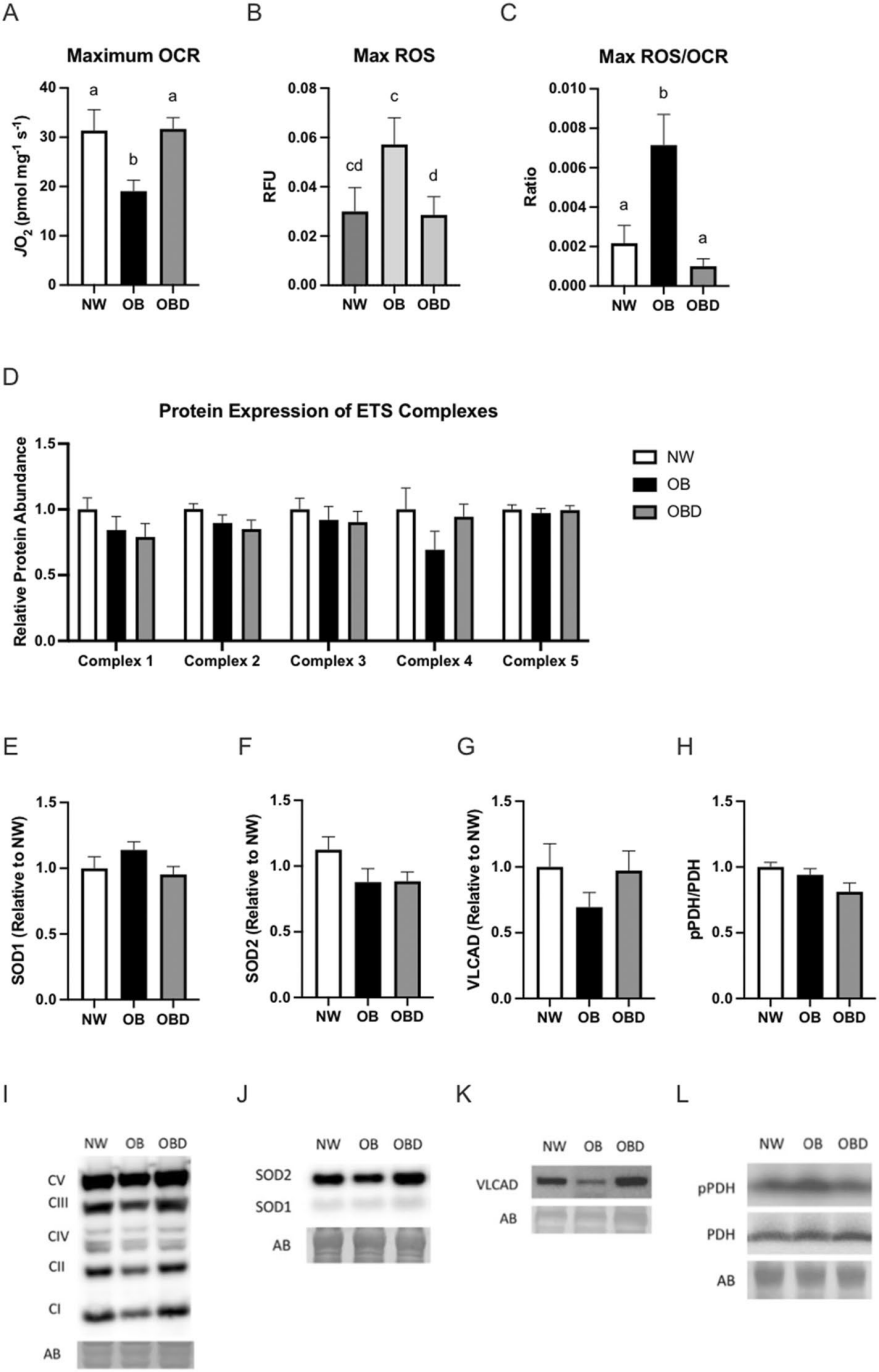
Skeletal muscle mitochondrial function. High-resolution respirometry and immunoblotting for selected protein expression was performed in skeletal muscle tissue collected 12 weeks after normal weight (NW), obese (OB) or obese diet supplemented (OBD). Skeletal muscle was obtained by biopsy of the semitendinosus muscle before being permeabilized and analyzed using an Oroboros O2K high-resolution respirometer for (**a**) mitochondrial oxidative capacity in the presence of metabolic substrates, (**b**) reactive oxygen species production, and (**c**) reactive oxygen species production relative to oxidative capacity. Muscle was used for immunoblotting of (**d**) electron transport system complexes I-V, (**e**, **f**) superoxide dismutase isoforms, (**g**) very long chain acyl-CoA dehydrogenase, and (**h**) phosphorylation of pyruvate dehydrogenase relative to NW. Representative western blots for (**i**) electron transport system complexes, (**j**) superoxide dismutase isoforms, (**k**) very long chain acyl-CoA dehydrogenase, and (**l**) phosphorylation of pyruvate dehydrogenase; ^ab^ Superscripts indicate differences at *p* < 0.05. Bars represent mean ± SEM.

Analysis of skeletal muscle mitochondrial function was performed using the Oroboros high-resolution respirometer (Oroboros Instruments, Innsbruck, AT) to determine the effect of obesity and dietary nutritional supplementation on maximum mitochondrial oxygen consumption rate (OCR) and reactive oxygen species (ROS) production. The maximum OCR was lower (*p* < 0.01) in skeletal muscle from OB than from NW or OBD (Fig. 5a). ROS release rates tended to be higher (*p* < 0.1) in OB compared to OBD, and were > twofold higher (*p* < 0.003) in OB than NW and OBD when expressed relative to OCR (Fig. 5b,c). Despite these functional differences, protein abundance of superoxide dismutases 1 and 2, very long chain acyl-CoA dehydrogenase, electron transport chain complexes, and the phosphorylation state of pyruvate dehydrogenase (PDH) were similar among groups (Fig. 5d–l).

### Short-term L-carnitine supplementation on insulin sensitivity

**Figure 6:**
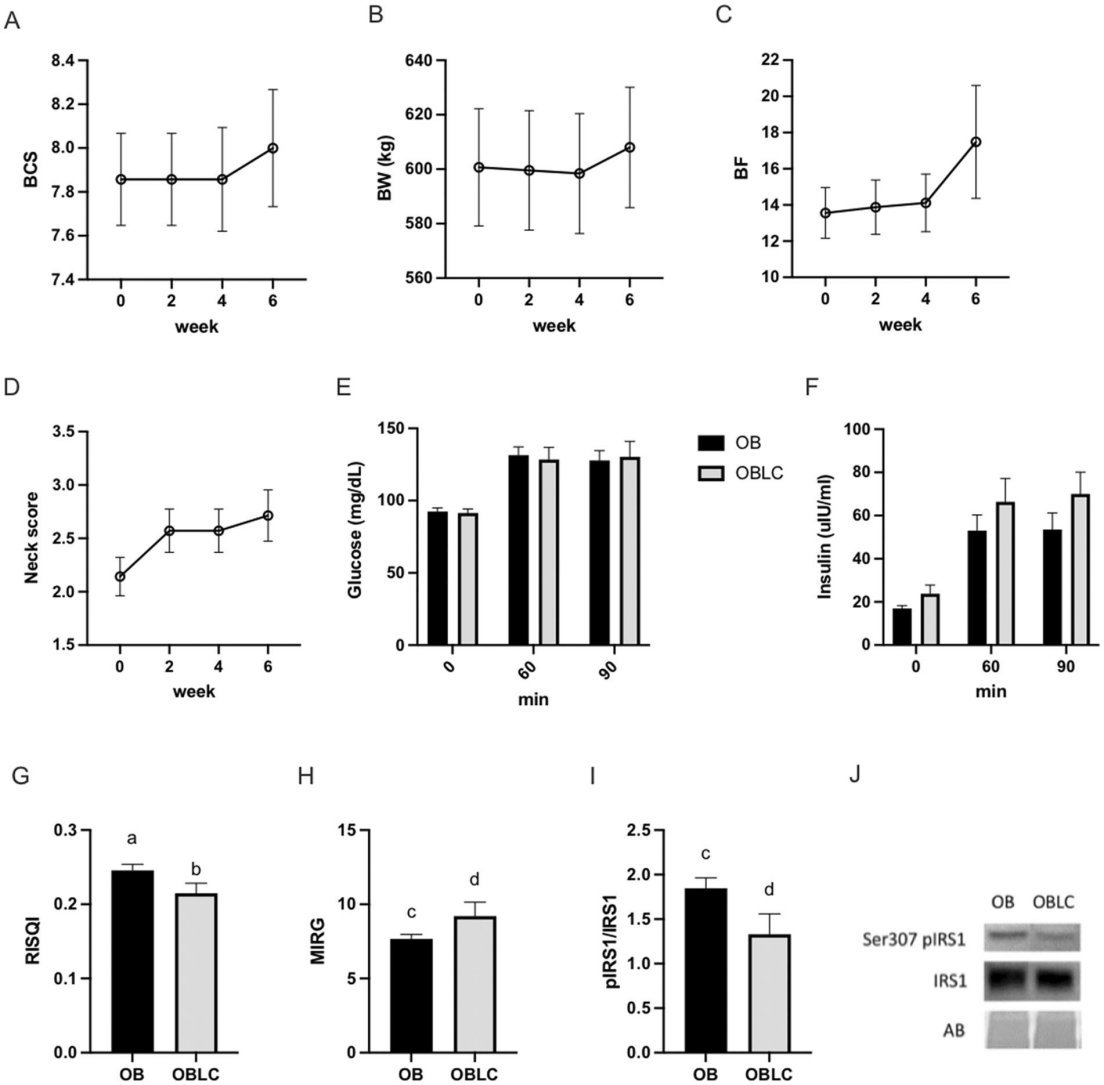
Morphometric and metabolic classification of mares during L-carnitine supplementation. Morphometric measurements and assessments of insulin dysfunction were taken at 2-week intervals before and during L-carnitine supplementation to obese mares including (**a**) body condition score (BCS), (**b**) body weight (BW), (**c**) percentage of body fat (BF) calculated using a tailhead fat measurement, (**d**) and cresty neck score (NS). Glucose (**e**) and insulin (**f**) concentrations response within group after oral administration light corn syrup for an oral sugar test at 0 (basal), 60- and 90-min. Proxies, including (**g**) reciprocal of the square root of insulin (RISQI) and (**h**) modified insulin to glucose ratio (MIRG), used to quantify insulin sensitivity and pancreatic response to glucose, respectively; (**i**) Ratio of phosphorylated IRS-1 to total IRS-1 in skeletal muscle; (**j**) Representative western blot for phosphorylation of IRS-1 to total IRS-1; ^ab^ Superscripts indicate differences at *p* < 0.05. ^cd^ Superscripts indicate differences at *p* < 0.1. Graphs represent mean ± SEM.

To determine the specific effects of L-carnitine after the completion of the above experiment, mares previously in the OB group were maintained on the same feeding regime with the exception of inclusion of dietary L-carnitine (OBLC) for 6 weeks before additional sample collections. No significant differences were observed in morphometric measurements before and after L-carnitine supplementation (Fig. 6a–d). Insulin regulation, as determined by an oral sugar test, was not affected (Fig. 6e,f); six out of the seven mares were diagnosed with insulin dysfunction prior to (OB) and after L-carnitine (OBLC). However, insulin sensitivity determined by RISQI was reduced (*p* = 0.01) after 6 weeks of L-carnitine consumption (Fig. 6g). Insulin response determined by MIRG tended (*p* = 0.09) to be higher for OBLC than OB, and expression of serine-307 phosphorylated insulin receptor substrate-1 (IRS-1) to total IRS-1 in skeletal muscle tissue was not different before and after L-carnitine supplementation (Fig. 6h–j).

### Fatty acid metabolism after short-term L-carnitine

**Figure 7:**
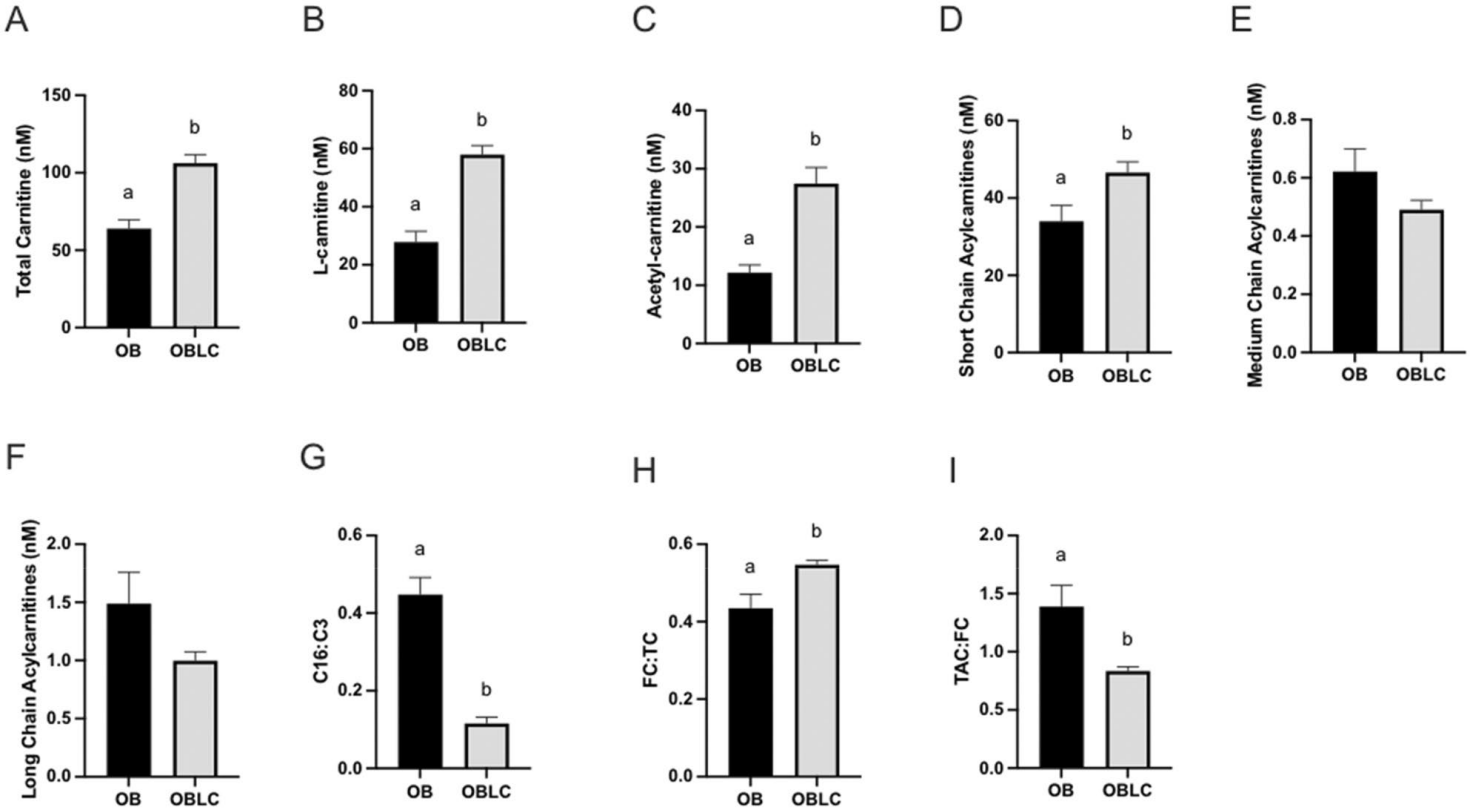
Acylcarnitine profile summary following 6 weeks of L-Carnitine. Serum samples were incubated and processed with an internal standard of known concentrations of acylcarnitines and analyzed using liquid chromatography mass spectrometry for obese mares before (OB) and after (OBLC) 6 weeks of L-carnitine supplementation for (**a**) total carnitine, (**b**) L-carnitine, and (**c**) total acetyl carnitines including (**d**) short chain, (**e**) medium chain, and (**f**) long chain. The ratio of (**g**) C16:C3 ratio was used to approximate completeness of β-oxidation as a measure of lipid metabolism efficiency, and ratios of (**h**) free carnitine to total carnitines or (**i**) total acetyl carnitines to free carnitine were used to compare carnitine availability. ^ab^ Superscripts indicate differences at *p* < 0.05. Graphs represent mean ± SEM.

Plasma acylcarnitines were evaluated after 6 weeks of L-carnitine supplementation to obese mares. As expected, total carnitine concentration (*p* = 0.004, Fig. 7a) and L-carnitine concentration (*p* = 0.0007, Fig. 7b) were greater following supplementation, demonstrating an uptake of L-carnitine. Overall, total acylcarnitines were elevated (*p* = 0.005) after supplementation (Fig. 7c). Analysis of individual species demonstrated that short chain acylcarnitines were more consistently increased (*p* = 0.05) than medium and long chain acylcarnitines (Fig. 7d–f, Table 2). The C16:C3 fatty acid ratio was lower following L-carnitine supplementation (*p* = 0.0004, Fig. 7g), suggesting an improvement in complete β-oxidation of long-chain fatty acids. After supplementation of L-carnitine, free carnitine relative to total carnitine was higher (*p* = 0.02, Fig. 7h), while total acylcarnitines compared to free carnitines decreased (*p* = 0.01, Fig. 7i).

**Table 2:** Concentrations (nM, mean ± SEM) of individual acylcarnitine species in plasma of obese mares (n = 7) before (OB) and after 6 weeks of L-carnitine oral supplementation (OBLC); ^ab^ Superscripts within the same row indicate difference at *p* < 0.05. ^cd^ Superscripts within the same row indicate tendency at *p* = 0.08.

	OB	OBLC
Short-chain acylcarnitines		
Acetyl (C2)	12.13 ± 1.34^a^	27.46 ± 2.76^b^
Propionyl (C3)	0.76 ± 0.09^a^	2.54 ± 0.28^b^
Succinyl (C4-DC)	18.99 ± 3.83	12.68 ± 1.47
Hydroxybutyryl (C4-OH)	0.39 ± 0.15	0.90 ± 0.24
Butanoyl (C4)	1.38 ± 0.27	2.29 ± 0.46
Hydroxyisovaleryl (C5-OH)	0.08 ± 0.02	0.12 ± 0.01
Isovaleryl (C5)	0.24 ± 0.04^a^	0.59 ± 0.04^b^
Medium-chain acylcarnitines		
Adipyl (C6-DC)	0.56 ± 0.09^a^	0.42 ± 0.07^b^
Hexanoyl (C6)	0.04 ± 0.01	0.05 ± 0.01
Decanoyl (C10)	0.05 ± 0.01	0.04 ± 0.01
Dodecanoyl (C12)	0.05 ± 0.01	0.04 ± 0.003
Long-chain acylcarnitines		
Tetradecenoyl (C14:1)	0.10 ± 0.01	0.08 ± 0.01
Tetradecanoyl (C14)	0.06 ± 0.01	0.05 ± 0.01
Hexadecenoyl (C16:1)	0.14 ± 0.03^a^	0.08 ± 0.02^b^
Palmitoyl (C16)	0.42 ± 0.09	0.31 ± 0.02
Linoleoyl (C18:2)	0.11 ± 0.01^a^	0.07 ± 0.01^b^
Octadecenoyl (C18:1)	0.67 ± 0.12^c^	0.41 ± 0.03^d^

### Mitochondrial function in skeletal muscle after L-carnitine supplementation

**Figure 8:**
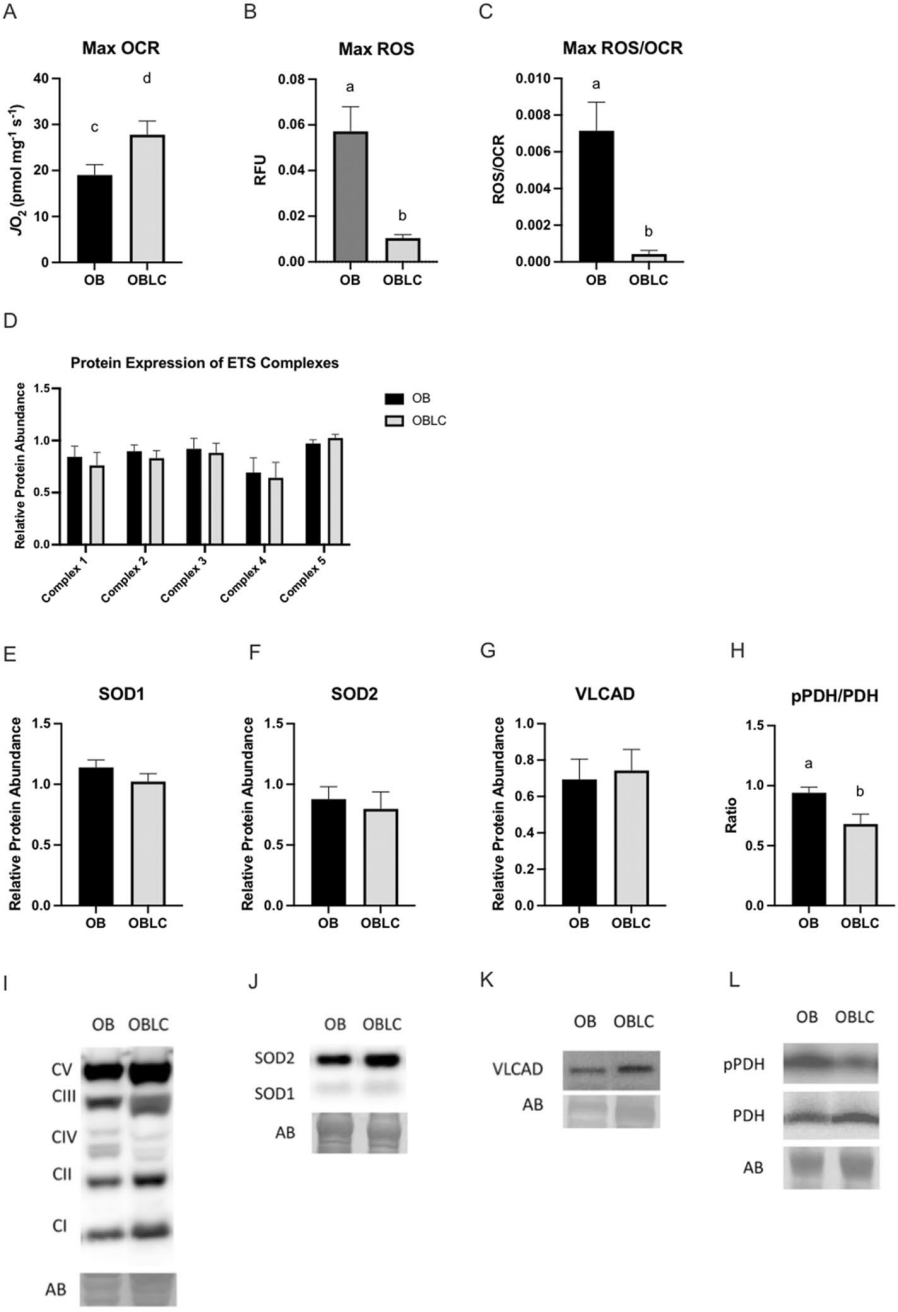
Skeletal muscle mitochondrial function. High-resolution respirometry and immunoblotting for selected protein expression was performed in skeletal muscle from obese mares before (OB) and after 6 weeks of dietary L-carnitine (OBLC). Skeletal muscle biopsies were taken from the trapezius muscle and were permeabilized and added to an Oroboros O2K high-resolution respirometer to determine (**a**) mitochondrial oxidative capacity in the presence of metabolic substrates, (**b**) reactive oxygen species production, and (**c**) reactive oxygen species production relative to oxidative capacity. Muscle was used for immunoblotting of (**d**) electron transport system complexes I-V, (**e**, **f**) superoxide dismutase isoforms, (**g**) very long chain acyl-CoA dehydrogenase, and (**h**) phosphorylation of pyruvate dehydrogenase relative to NW. Representative western blots for (**i**) electron transport system complexes, (**j**) superoxide dismutase isoforms, (**k**) very long chain acyl-CoA dehydrogenase, and (**l**) phosphorylation of pyruvate dehydrogenase. ^ab^ Superscripts indicate differences at *p* < 0.05. ^cd^ Superscripts indicate differences at *p* < 0.1. Graphs represent mean ± SEM.

A skeletal muscle biopsy was taken after 6 weeks of L-carnitine supplementation to obese mares. After L-carnitine supplementation, maximum OCR tended to be greater (*p* = 0.09, Fig. 8a) and ROS production was lower (*p* < 0.005, Fig. 8b,c). Protein abundance of SOD1, SOD2, or electron transport chain complexes did not change after L-carnitine supplementation (Fig. 8d–g,i–k), while phosphorylation of PDH was decreased (*p* = 0.02; Fig. 8h,l).

## Discussion

In mares, limited research has been conducted to specifically assess insulin dysfunction and how dietary modifications can affect their metabolic and reproductive status. In the present study, we assessed the effects of adiposity on metabolic status, skeletal muscle mitochondrial function, insulin signaling, and lipid metabolism in mares and we examined the potential for dietary supplements to improve the metabolic function of skeletal muscle and systemic insulin dysregulation.

Cellular insulin sensitivity is not currently recognized as a defining characteristic in insulin-dysregulated horses; however, gastrointestinal factors such as incretin hormone release and overall gastrointestinal glucose uptake are known to be altered in insulin-dysregulated horses^36^. To date, the specific mechanisms that drive cellular insulin resistance in equine skeletal muscle are not understood. We have shown for the first time that serine-307 phosphorylation of insulin receptor substrate 1 (IRS1) is higher in skeletal muscle collected from obese mares with insulin dysregulation. This posttranslational modification is characteristic of insulin resistance in humans, which prevents the uptake of glucose into myocytes in response to insulin^22^. In addition, this signaling can be disrupted in humans by inflammation and/or oxidative stress, resulting in the phosphorylation of IRS1 and subsequent inhibition of GLUT-4 translocation, reducing cellular glucose uptake^37^. The IRS1 phosphorylation could serve as a cellular marker to identify insulin-dysregulated horses in inconclusive cases. However, the mechanisms responsible for IRS1 phosphorylation in horses require further investigation. The MIRG proxy suggested increased pancreatic insulin release in the obese groups, which is consistent with prediabetes in humans and insulin dysregulation in horses, as beta cells initially produce excessive insulin in order to reduce blood glucose^36,38^.

Skeletal muscle is the primary consumer of glucose in mammals^39^, affecting systemic glucose regulation^16^. We evaluated the effects of obesity on mitochondrial function, lipid metabolism, and oxidative stress in live skeletal muscle cells. Skeletal muscle from obese mares had lower mitochondrial oxidative capacity and greater ROS production compared to normal-weight mares, consistent with mitochondrial dysfunction^40^. ROS are capable of damaging lipids, proteins, and DNA unless removed by antioxidant enzymes or molecules^41–43^. In the present study, mitochondrial or cytoplasmic superoxide dismutase (SOD) protein expression was not altered in obese mares. Similar to our results, antioxidant parameters in equine skeletal muscle such as glutathione, activity of glutathione peroxidase (GPX), and expression of *GPX*, catalase, peroxiredoxin, glutathione synthetase, and glutathione reductase were not altered with adiposity. Only total superoxide dismutase activity was upregulated with increasing adiposity^23^. No oxidative damage was observed in skeletal muscle related to obesity alone or associated with hyperinsulinemia, suggesting that oxidative damage linked to obesity in skeletal muscle is not essential for the pathogenesis of hyperinsulinemia in obese horses^23^. However, ROS production in equine skeletal muscle is inversely associated with insulin sensitivity^23^. While we did not observe any decrease in mitochondrial protein expression to explain the lower OCR in obese mares, we did not evaluate changes in mitochondrial dynamics or individual enzyme activities that might have contributed to this effect^44^.

In mammals, including humans, lipid metabolism is closely coordinated with glucose homeostasis in response to insulin stimulation^16^. In humans, insulin resistance can also be induced by increased plasma concentrations of lipids^16^. Altered lipid metabolism is often characteristic of obesity, usually due to elevated circulating lipids and potentially impaired glucose uptake due to insulin resistance^45^. Metabolic dysregulation of plasmatic lipids promotes mitochondrial dysfunction due to increased β-oxidation and subsequent peroxidation^46^. In our study, circulating triglycerides and lipid composition of red blood cells were similar among groups and over time. In obese mares, omega-6 polyunsaturated fatty acid (n-6 PUFA) were increased, while EPA + DHA, omega-3 polyunsaturated fatty acids (n-3 PUFA), and non-esterified (NEFA) were reduced when compared to normal-weight mares. Lower NEFA in obese groups may reflect a possible mitigation of adipose tissue lipolysis following grain feeding, as carbohydrate intake has been previously demonstrated to reduce NEFA concentrations in humans^47^, although a similar study has not been done in horses and the cause of altered NEFA concentrations was not determined in this study. The altered fatty acid distribution in obese mares was possibly caused by the adiposity associated with high caloric intake due to the inclusion of grain rather than dietary fatty acids. In humans, n-3 PUFAs have been demonstrated to reduce the production of inflammatory mediators, resulting in a positive effect on obesity, insulin resistance, and mitochondrial dysfunction^48,49^. There is a debate about whether n-6 PUFAs have pro- or anti-inflammatory effects^48^. In a mouse model, high levels of n-6 PUFAs promote the reduction of IRS1 phosphorylation in skeletal muscle^50^, which may exacerbate insulin resistance. Overall, insulin dysfunction is a complex syndrome that disrupts the close association between lipid metabolism and glucose homeostasis. Characterization of these mechanisms in obese horses can aid to identify new therapeutic approaches to reduce the negative effects of insulin dysfunction.

The most common treatments to improve obesity-related metabolic disorders in horses and people involve weight loss through dietary restrictions and exercise^16,25,26^. In horses, diet and exercise programs need to be adapted to the individual’s demands for weight loss and improvement of insulin sensitivity, but such programs can have potential complications in certain horses^25,51^. Specific dietary supplements have been shown to improve insulin sensitivity in humans and mice, and they have also been studied in horses. Supplementation with prebiotics such as short-chain fructo-oligosaccharides into the diet of obese horses has no effect on systemic glucose, triglycerides, or leptin, although they improve insulin sensitivity^52^; this beneficial effect is enhanced by the addition of vitamin and mineral nutraceutical supplements^53^. Similarly, the synergetic action of dietary leucine and resveratrol in insulin-dysregulated horses promotes an increase in insulin sensitivity and high molecular-weight adiponectin, an insulin-sensitizing adipokine^54^. In the present study, obese mares were fed a dietary supplement formated to improve metabolism and gastrointestinal health in an attempt to restore insulin signaling and cellular function. When provided the targeted dietary intervention, obese mares had better glucose tolerance and insulin sensitivity, similar to normal-weight mares. In addition, skeletal muscle from diet supplemented obese mares (OBD) exhibited insulin signaling changes, as the abundance of serine-307 phosphorylated IRS1 was reduced to a similar level as in normal-weight mares. These findings support the concept that complex dietary supplementation can improve insulin dysregulation by targeting cellular mechanisms in the absence of exercise or weight loss in horses. The potential for dietary interventions to improve insulin resistance warrants further investigation.

Metabolic compounds such as L-carnitine have been used as dietary supplements alone or in combination to aid metabolic disorders. L-carnitine is the only molecule capable of transporting long-chain fatty acids across the inner mitochondrial membrane, where they are metabolized into short-chain fats, as β-oxidation cleaves two carbons per cycle to form a single acetyl-CoA molecule that can enter the citric acid cycle to produce ATP^55,56^. Acylcarnitines are the esters formed when fatty acids bind to L-carnitine to be carried into the mitochondria; thus, analysis of acylcarnitine species are indicative of fat uptake and metabolism in the mitochondria^55,56^. Obese mares provided the complex diet supplement (OBD) resulted in increased circulating free L-carnitine and total acetylcarnitine species with a larger proportion of short-chain than medium- or long-chain acylcarnitines, implying completeness of β-oxidation due to improved mitochondrial function and fatty acid metabolism^56^. We subsequently examined the isolated effect of dietary L-carnitine on insulin dysregulation and lipid metabolism in obese mares (OBLC). Dietary L-carnitine did not result in changes in body condition of obese mares, suggesting it did not alter adiposity. However, proxies evaluating systemic insulin response tended to improve, and the ratio of phosphorylated IRS1 tended be less in skeletal muscle, suggesting a potential influence on insulin sensivity. However, supplementation with L-carnitine alone lacked the significant impact observed with the complex diet supplementation, suggesting that synergistic interactions among the nutrients were important to optimize beneficial effects. As expected, dietary L-carnitine alone promoted higher systemic free L-carnitine and total acetylcarnitines, predominantly short-chain acylcarnitines. In humans, dietary L-carnitine increased plasmatic levels of free L-carnitine and acetylcarnitines and was associated with lower glucose and insulin in insulin-resistant individuals^28^. In overweight or obese women with polycystic ovary syndrome subjected to L-carnitine supplementation, insulin sensitivity was improved but lipid profiles were not altered^57^. Importantly, a meta-analysis of random trials demonstrates that dietary L-carnitine is effective in treating people with insulin resistance, and that the benefits increase with longer consumption^58^. Consistent with our study, dietary L-carnitine increased plasmatic free L-carnitine and short-chain acylcarnitines in 3-year old horses but did not improve insulin sensitivity^59^, possibly because L-carnitine effects are more evident in insulin-dysregulated horses as observed in insulin-sensitive humans^28^. Dietary supplementation combining the synergistic effects of vitamins, trace minerals, amino acids (including L-carnitine), antioxidants, omega-3 fatty acids, prebiotics and probiotics appears promising to promote metabolic changes and improve mitochondrial function and lipid metabolism in obese patients.

Dietary strategies have been used in an attempt to modulate metabolic disorders; these include antioxidants compounds. Several antioxidants such as lipoic acid, resveratrol, coenzyme Q10 and epigallocatechin‐3‐gallate have anti‐inflammatory properties and improve cellular glucose metabolism by reducing levels of oxidative stress^60,61^. In our study, obesity was associated with lower mitochondrial oxygen consumption in skeletal muscle and higher ROS production. Normal-weight and obese mares provided the diet supplement (OBD) had similar levels of skeletal muscle oxygen consumption and ROS production. Dietary L-carnitine alone promoted a reduction of ROS production in the skeletal muscle of obese mares. However, abundance of antioxidant enzymes such as SOD1 and SOD2, and electron transport chain complexes in skeletal muscle were not affected by obesity or dietary supplements. This finding may be explained by L-carnitine acting as an antioxidant to prevent lipid buildup and peroxidation in mitochondria^62,63^. Also, exogenous antioxidants contained in the dietary supplement may have either supplied the necessary antioxidant capacity or improved the endogenous potential to reduce oxidant production. For instance, lipoic acid is a potent antioxidant and a cofactor of mitochondrial dehydrogenase complexes, which can directly scavenge ROS or interact with other antioxidant molecules such as coenzyme Q10, vitamin C, and vitamin E to indirectly reduce oxidative stress^61^. Interestingly, we observed that dietary L-carnitine was associated with reduced phosphorylation of pyruvate dehydrogenase (PDH). Phosphorylation downregulates PDH activity to metabolize pyruvate into acetyl-CoA in mitochondria^64^. Similar responses have been observed in mice and insulin-resistant patients subjected to dietary L-carnitine supplementation, showing increased PDH activity in skeletal muscle^27,28^. Overall, dietary antioxidants and L-carnitine appeared to reduce ROS production in the skeletal muscle independently of antioxidant mitochondrial enzymes and may have an impact on pyruvate metabolism for energy production in obese mares.

In summary, obesity was associated with insulin dysregulation, oxidative stress, and decreased mitochondrial function in mares. Results of the present study demonstrated that targeted dietary supplementation can reverse systemic and cellular negative effects associated with obesity, independent of weight loss or exercise, with the potential to improve systemic health. Additional research is needed to elucidate the mechanisms of insulin dysregulation and mitochondrial dysfunction in obese horses to create novel therapies and further improve well-being. In addition, specifically formulated dietary supplementation may provide support for the treatment of insulin resistance and diabetes in humans. Future studies are needed to address the role of inflammatory mechanisms in inducing cell stress and mitochondrial dysfunction to determine specific dietary components to restore cell function.

## Methods

### Study population and serum collection

Animal procedures were approved by Colorado State University’s Institutional Animal Care and Use Committee and were carried out in accordance with relevant guidelines and regulations. All methods are reported in accordance with ARRIVE guidelines. Prior to start of the study, nonlactating, light-horse mares were evaluated for body condition scores (BCS, from 1, emaciated to 9, extreme obesity)^65^, and divided into three groups based on age. All mares were on a hay only diet prior to the start of the study, although they came from different locations. All mares had a BCS from 5 to 8 prior to assignment into groups. Mares with an initial BCS of 5 or 6 (moderate to moderately fleshy BCS) were assigned to the normal-weight group (NW, n = 6, 17.8 ± 1.8 years, BCS 5.4 ± 0.2, mean ± SD). The remaining mares had BCS from 6 to 8 (moderately fleshy to fat), were paired by age, and then randomized into one of two overweight groups: obese (OB, n = 7, 18.6 ± 1.5 years, BCS 7.2 ± 0.3) and obese dietary supplemented (OBD, n = 7, mean age of 17.7 ± 1.4 years, BCS 6.9 ± 0.3). Mares were housed in large dry lots and each group was provided a unique dietary regime for the entirety of the study. Mares grouped as NW were fed grass/alfalfa mix hay at approximately 2% of body weight daily and 57 g daily of a commercial forage balancer (Free Balance 12:12®, Purina Animal Nutrition, Gray Summit, MO, USA). The base diet for OB and OBD was grass/alfalfa hay ad libitum and forage balancer. Twice daily, OB and OBD were also fed 0.75 kg of whole oats and 0.75 kg of cracked corn provided in a single lot from a local feed supplier. Mares in the OBD group received targeted supplementation designed to support equine metabolic and gastrointestinal health (187 g, divided into two feedings), consisting of a combination of vitamins, trace minerals, amino acids (including L-carnitine), antioxidants, omega-3 fatty acids, prebiotics and probiotics formulated to support cellular metabolism (Platinum Performance Inc., Buellton, CA, USA). Mares were maintained on group-specific feeding regimens for approximately 12 weeks before blood and muscle collections.

In a follow-up study aimed to assess the isolated effects of L-carnitine supplementation , mares that were originally in the OB group were kept on the same diet (ad libitum hay, daily grain and forage balancer), but they were also fed 7.5 g of L-carnitine powder twice daily for 6 weeks, which was equivalent to the amount of L-carnitine included in the dietary supplement for OBD. The L-carnitine supplemented obese mares (OBLC) were evaluated biweekly for markers of adiposity, as described below, for 6 weeks prior to blood and muscle collections. The effect of L-carnitine supplementation to the obsese mares was determined by comparing samples collected at 12 weeks for OB to samples collected from the same mares after an additional 6 weeks of L-carnitine supplementation, designated as OBLC. An visual representation of the experimental design is provided in Supplemental Figure S2.

### Morphometric and insulin dysfunction assessments

Morphometric assessments were performed at 2-week intervals and included BCS (1–9)^65^ and cresty neck score (0–5)^66^. An approximation of the percentage of body fat was calculated using a measurement of subcutaneous fat thickness obtained using a 10 MHz linear-array transducer that was placed approximately 7.6 cm cranial and 5 cm lateral from the tailhead and calculated using the following formula [(2.47 + 5.47 × tailhead fat (cm)]^67,68^.

Insulin dysregulation was evaluated using an oral sugar test. Mares were fasted for at least 6 h before being given an oral dose of Kroger® Lite Corn Syrup (The Kroger Co, Cincinnati, OH) at 0.15 mL/kg body weight^34^. Blood samples were collected into EDTA tubes by jugular venipuncture prior to and at 60 and 90 min after administration of corn syrup. Blood glucose was measured from whole blood directly after collection using a blood glucose monitor equilibrated for equine blood following the manufacturer’s instructions (AlphaTRAK 2, Zoetis Inc., Kalamazoo, MI). For insulin measurements, blood was allowed to clot for 2 h at 4 °C, centrifuged at 1500 g for 10 min, and plasma was stored at − 20 °C until radioimmunoassays for insulin quantification by a reference lab (Endocrinology Lab, Cornell University Animal Health Diagnostic Center, Ithaca, NY). Insulin concentrations at either 60- or 90-min timepoints that were greater than 45 µU/mL were considered indicative of insulin dysregulation^35^. Insulin and glucose values collected prior to sugar administration were used to calculate the reciprocal of the square root of insulin [RISQI; (fasted insulin)^-0.5^] and the modified insulin-to-glucose ratio [MIRG; 800 − 0.3 (fasted insulin − 50)^2^ / (fasted glucose − 30)], with the proxies estimating insulin sensitivity and pancreatic response to glucose, respectively^69^.

### Red blood cell lipid analysis

Before and at 4 and 8 weeks of dietary supplementation, 10 mL of whole blood was collected into EDTA tubes and centrifuged at 3000 g for 10 min at room temperature. Pelleted red blood cells were transferred into cryovials, snap frozen in liquid nitrogen, and shipped on dry ice for membrane fatty acid composition (OmegaQuant Analytics LLC, Sioux Falls, South Dakota). Red blood cell membrane lipids were transesterified with boron trifluoride prior to gas chromatography to determine fatty acid composition, expressed as a weight percentage of total identified fatty acids^70^.

### Determination of circulating non-esterified free fatty acids, triglycerides, and acylcarnitine species

Plasma samples were collected from all mares after 12 weeks of their respective (NW, OB, and OBD) diet, and again after 6 weeks of dietary L-carnitine for OBLC. Blood was collected by jugular venipuncture into EDTA tubes and centrifuged at 3000 g prior to plasma collection. Plasma was snap frozen and stored at − 20 °C until analysis. Plasma concentrations of non-esterified free fatty acids were determined by a reference laboratory (Clinical Pathology Laboratory, Cornell University Animal Health Diagnostic Center). Plasma triglyceride concentrations were determined using a colorimetric assay kit (Cayman Chemical, Ann Harbor, MI, USA) using a single non treated 96-well microplate, read at 540-nm absorbance on a Synergy 2 microplate reader (Biotek, Agilent, Santa Clara, CA, USA). Plasma acylcarnitine profiles were assessed using liquid chromotography mass spectrometry at an external laboratory (University of Colorado Anschutz Medical Campus School of Medicine Metabolomics Core, Aurora, CO)^71^. Briefly, a standard solution (180 uL) containing known concentrations of acylcarnitine species in methanol were added to 20-µL aliquots of plasma. Samples were briefly vortexed and incubated at 20 °C for 15 min, then centrifuged at 12700 g. Approximately 100 µL of supernatant was transferred to a new tube, and 100 µL of 10 mM ammonium acetate was added to each sample. A 10-uL allotment of each sample was injected and analyzed using a Thermo Vanquisher Liquid Chromatography system coupled to a Q-Exactive™ Quadrupole-Orbitrap™ Mass Spectrometer as previously described in detail^71^.

### Muscle sample collection

Punch biopsies of the semitendinosus muscle were obtained after mares were on their assigned diet for 12 weeks. Samples were immediately washed in BIOPS preservation solution containing 10 mM Ca-EGTA (0.1 M free calcium), 20 mM imidazole, 20 mM taurine, 50 mM K-MES, 0.5 mM DTT, 6.56 mM MgCl_2_, 5.77 mM ATP, and 15 mM phosphocreatine at pH 7.1^72^. Half of the sample was snap frozen in liquid nitrogen and stored at − 80 °C for western blot analysis, while the other half was placed in fresh BIOPS buffer on ice for high-resolution respirometry.

### High-resolution respirometry

Skeletal muscle samples were stored on ice overnight in BIOPS. Next day, samples were teased and treated with 50 µg/mL saponin on ice for 20 min with gentle rocking to permeabilize cell membranes while leaving mitochondrial membranes intact. Permeabilized fibers were then removed and rinsed in mitochondrial respiration medium (MIR05; 0.5 mM EGTA, 3 mM MgCl_2_ hexahydrate, 60 mM lactobionic acid, 20 mM taurine, 10 mM KH_2_PO_4_, 20 mM HEPES, 110 mM sucrose, and 0.1% BSA; pH 7.1). Fibers were gently blotted dry using Whatman® filter paper (Cytiva, Marlborough, MA) and weighed immediately prior to adding approximately 5 mg to the oxygraph chamber containing 2 mL MIR05, horseradish peroxidase (1 U/mL), and Amplex UltraRed (5 µM) at 37 °C. The chambers were saturated with > 400 µM oxygen and stabilized before addition of metabolic substrates in order to maintain an assay oxygen concentration between 300–400 µM to avoid diffusion limitation on OCR and ROS release rates^72^. Maximum mitochondrial oxidative capacity (OCR) was evaluated following the addition of 1 mM malate, 0.05 mM palmitoylcarnitine, 5 mM pyruvate, 2.5 mM adenosine diphosphate, and 10 mM succinate. The net rate of mitochondrial H_2_O_2_ (ROS) release was measured simultaneously with OCR by monitoring the accumulation of chamber resorufin (Ex/Em 571/585 nm), the stable fluorescent product of 1:1 oxidation of Amplex UltraRed by H_2_O_2_ in the presence of horseradish peroxidase (HRP; 1 U/mL) following the addition of succinate using a custom-fitted fluorometer (O2k-Fluo LED2 module; Oroboros Instruments, Innsbruck, AT)^72–74^.

### Protein sample preparation and immunoblotting

Antibodies were purchased in Abcam (Waltham, MA), unless otherwise stated. Primary antibodies were phospho-IRS1 (Ser307), IRS1, OXPHOS, Cu/ZN SOD (SOD101; StressGen, Victoria, BC), Mn SOD (SOD111; StressGen), VLCAD (Santa Cruz Biotechnology, Dallas, TX), phospho-PDH, and PDH. Secondary antibodies included goat anti-rabbit IgG H&L-HRP and goat anti-mouse IgG H&L-HRP.

Frozen skeletal muscle tissue was thawed on ice, chipped to attain 30 mg of sample, and homogenized in M-PER™ Mammalian Protein Extraction Reagent lysis buffer (Thermo Fisher Scientific, Waltham, MA) containing Halt™ Protease and Phosphatase Inhibitor Single-Use Cocktail (Thermo Fisher Scientific) using a glass tissue homogenizer. Samples were sonicated and centrifuged at 10000 g for 15 min. The supernatant was transferred into a new microcentrifuge tube and stored at − 80 °C. Concentrations of protein were determined using the Pierce™ bicinchoninic acid assay (Thermo Fisher Scientific). Protein samples were prepared with Laemmli Buffer (Bio-Rad, Hercules, CA) and DTT Bolt™ (Thermo Fisher Scientific) to achieve concentrations of 30 µg/mL. Samples were denatured for 10 min at 90 °C, cooled, and loaded into 12-well, 4–12% Bis–Tris polyacrylamide gels (Thermo Fisher Scientific) with a protein ladder to determine weight (Precision Plus Protein Standards Dual Color, Bio-Rad). Gels were run for 45 min at 150 V, removed, and protein was transferred to a polyvinylidene difluoride (PVDF) membrane. The resulting membrane was washed in Tris-buffered saline (TBS) + Tween® (Sigma-Aldrich, St Louis, MO) and blocked with 5% non-fat dry milk in TBS for 1 h. Following a wash, 1:1000 primary antibody in milk-TBS solution was added and rocked overnight at 4 °C. After washing, 1:3000 secondary antibody was added and rocked for 1 h. The membrane was washed and imaged with chemiluminescence. Band density representing the protein of interest’s molecular weight were quantified via colorimetric analysis using ImageJ (NIH, Bethesda, MD) and standardized to total protein concentrations determined using Amido Black (Bio-Rad, Hercules, CA) protein stain^75^.

### Statistical analysis

Statistical analyses were conducted using GraphPad Prism 9.3.1 software. Continuous datasets were analyzed for normality and homogeneity of variances using the Shapiro–Wilk and Levene test. Repeated measures of morphometric data and glucose tolerance tests were compared within and between groups using two-way ANOVA with post-hoc Tukey’s multiple comparison tests. Western blots, acylcarnitine profiles, oral sugar tests, and high resolution respirometry results were analyzed by one-way ANOVA with post-hoc Tukey’s multiple comparison tests for normally distributed data sets. Kruskal–Wallis tests, followed by Dunn’s multiple comparison tests, were used for data that failed to be normally distributed. All pre-post analyses, before and after L-carnitine supplementation, were evaluated using two-tailed paired t-test. Values of *p* < 0.05 were considered significant, while values *p* ≤ 0.1 were considered tendencies. Results are presented as mean ± SEM.

### Supplementary Information

Supplementary Figures.

Supplementary Information 2.

## Data Availability

The data generated during and/or analyzed in the current study are available from the corresponding authors upon reasonable request.

## References

[1] Giles SL, Rands SA, Nicol CJ, Harris PA (2014). Obesity prevalence and associated risk factors in outdoor living domestic horses and ponies. PeerJ.

[2] Kosolofski, H. R., Gow, S. P. & Robinson, K. A. Prevalence of obesity in the equine population of Saskatoon and surrounding area. *Can. Vet. J.* **58** PMC5556474 28878421

[3] Ertelt A, Barton A-K, Schmitz RR, Gehlen H (2014). Metabolic syndrome: Is equine disease comparable to what we know in humans?. Endocr. Connect..

[4] Rendle D (2018). Equine obesity: current perspectives. UK-Vet. Equine.

[5] Johnson PJ, Wiedmeyer CE, LaCarrubba A, Ganjam VKS, Messer NT (2012). Diabetes, insulin resistance, and metabolic syndrome in horses. J. Diabetes Sci. Technol..

[6] Morgan RA, Keen JA, Walker BR, Hadoke PWF (2016). Vascular dysfunction in horses with endocrinopathic laminitis. PLoS ONE.

[7] National Health Statistics Reports, Number 158, June 14, 2021 (2021).

[8] Rashid MN, Fuentes F, Touchon RC, Wehner PS (2003). Obesity and the risk for cardiovascular disease. Prev. Cardiol..

[9] Wondmkun YT (2020). Obesity, insulin resistance, and type 2 diabetes: Associations and therapeutic implications. DMSO.

[10] Aronow WS (2017). Association of obesity with hypertension. Ann. Transl. Med..

[11] Kottaisamy CPD, Raj DS, Prasanth Kumar V, Sankaran U (2021). Experimental animal models for diabetes and its related complications—A review. Lab. Anim. Res..

[12] de Mello AH, Costa AB, Engel JDG, Rezin GT (2018). Mitochondrial dysfunction in obesity. Life Sci..

[13] Schrauwen, P., Schrauwen-Hinderling, V., Hoeks, J. & Hesselink, M. K. C. Mitochondrial dysfunction and lipotoxicity. *Biochim. Biophys. Acta BBA Mol. Cell Biol. Lipids* **1801**, 266–271 (2010). 10.1016/j.bbalip.2009.09.011 19782153

[14] Siard-Altman MH (2020). Relationships of inflamm-aging with circulating nutrient levels, body composition, age, and pituitary pars intermedia dysfunction in a senior horse population. Vet. Immunol. Immunopathol..

[15] Sessions-Bresnahan DR, Heuberger AL, Carnevale EM (2018). Obesity in mares promotes uterine inflammation and alters embryo lipid fingerprints and homeostasis. Biol. Reprod..

[16] Li, M. *et al.* Trends in insulin resistance: Insights into mechanisms and therapeutic strategy. *Signal Transduct. Targeted Ther.* **7** (2022). 10.1038/s41392-022-01073-0 PMC9259665 35794109

[17] Stewart-Hunt L, Pratt-Phillips S, McCUTCHEON LJ, Geor RJ (2010). Dietary energy source and physical conditioning affect insulin sensitivity and skeletal muscle glucose metabolism in horses: Diet, insulin sensitivity and muscle glucose metabolism in horses. Equine Vet. J..

[18] Kayar SR, Hoppeler H, Mermod L, Weibel ER (1988). Mitochondrial size and shape in equine skeletal muscle: A three-dimensional reconstruction study. Anat. Rec..

[19] Kearns CF, McKeever KH, Abe T (2002). Overview of horse body composition and muscle architecture: Implications for performance. Vet. J..

[20] Tyrovolas S (2016). Factors associated with skeletal muscle mass, sarcopenia, and sarcopenic obesity in older adults: A multi-continent study: Sarcopena and sarcopenic obesity in older adults. J. Cachexia Sarcopenia Muscle.

[21] Yaribeygi H, Farrokhi FR, Butler AE, Sahebkar A (2019). Insulin resistance: Review of the underlying molecular mechanisms. J. Cell. Physiol..

[22] Waller AP, Burns TA, Mudge MC, Belknap JK, Lacombe VA (2011). Insulin resistance selectively alters cell-surface glucose transporters but not their total protein expression in equine skeletal muscle: Insulin resistance and glucose transport. J. Vet. Internal Med..

[23] Banse HE, Frank N, Kwong GPS, McFarlane D (2015). Relationship of oxidative stress in skeletal muscle with obesity and obesity-associated hyperinsulinemia in horses. Can. J. Vet. Res..

[24] Tangvarasittichai S (2015). Oxidative stress, insulin resistance, dyslipidemia and type 2 diabetes mellitus. WJD.

[25] Morgan RA, Keen JA, McGowan CM (2016). Treatment of equine metabolic syndrome: A clinical case series. Equine Vet. J..

[26] Argo CMCG (2012). Weight loss resistance: A further consideration for the nutritional management of obese Equidae. Vet. J..

[27] Noland RC (2009). Carnitine insufficiency caused by aging and overnutrition compromises mitochondrial performance and metabolic control. J. Biol. Chem..

[28] Muoio DM (2012). Muscle-specific deletion of carnitine acetyltransferase compromises glucose tolerance and metabolic flexibility. Cell Metab..

[29] Vincent JB (2017). new evidence against chromium as an essential trace element. J. Nutr..

[30] Jamilian M (2018). the influences of chromium supplementation on glycemic control, markers of cardio-metabolic risk, and oxidative stress in infertile polycystic ovary syndrome women candidate for in vitro fertilization: A randomized, double-blind. Placebo-Controlled Trial. Biol. Trace Elem. Res..

[31] Adeva-Andany MM, Calvo-Castro I, Fernández-Fernández C, Donapetry-García C, Pedre-Piñeiro AM (2017). Significance of L-carnitine for human health. IUBMB Life.

[32] Surai, P. F. Antioxidant Action of Carnitine: Molecular Mechanisms and Practical Applications (2015).

[33] Catandi GD (2022). Oocyte metabolic function, lipid composition, and developmental potential are altered by diet in older mares. Reproduction.

[34] Jocelyn NA, Harris PA, Menzies-Gow NJ (2018). Effect of varying the dose of corn syrup on the insulin and glucose response to the oral sugar test. Equine Vet. J..

[35] Frank, N. Laboratory Testing for Endocrine and Metabolic Disorders. in *Interpretation of Equine Laboratory Diagnostics* 401–408 (John Wiley & Sons, Inc., 2017). 10.1002/9781118922798.ch60.

[36] De Laat MA, McGree JM, Sillence MN (2016). Equine hyperinsulinemia: investigation of the enteroinsular axis during insulin dysregulation. Am. J. Physiol. Endocrinol. Metab..

[37] Abel ED, O’Shea KM, Ramasamy R (2012). Insulin resistance: Metabolic mechanisms and consequences in the heart. Arterioscler. Thromb. Vasc. Biol..

[38] Thomas DD, Corkey BE, Istfan NW, Apovian CM (2019). Hyperinsulinemia: An early indicator of metabolic dysfunction. J. Endocr. Soc..

[39] Merz, K. E. & Thurmond, D. C. Role of skeletal muscle in insulin resistance and glucose uptake. in *Comprehensive Physiology* (ed. Terjung, R.) 785–809 (Wiley, 2020). 10.1002/cphy.c190029. 10.1002/cphy.c190029 PMC8074531 32940941

[40] Zhao R, Jiang S, Zhang L, Yu Z (2019). Mitochondrial electron transport chain, ROS generation and uncoupling (review). Int. J. Mol. Med..

[41] Yu X, Long YC (2016). Crosstalk between cystine and glutathione is critical for the regulation of amino acid signaling pathways and ferroptosis. Sci. Rep..

[42] Ribas, V., García-Ruiz, C. & Fernández-Checa, J. C. Glutathione and mitochondria. *Front. Pharmacol.* **5** (2014). 10.3389/fphar.2014.00151 PMC4079069 25024695

[43] Henry, M. L. *et al.* The impact of n-acetyl cysteine and coenzyme q10 supplementation on skeletal muscle antioxidants and proteome in fit thoroughbred horses. *Antioxidants* **10** (2021). 10.3390/antiox10111739 PMC8615093 34829610

[44] Das, M., Sauceda, C. & Webster, N. J. G. Mitochondrial dysfunction in obesity and reproduction. *Endocrinology* **162**, bqaa158 (2021). 10.1210/endocr/bqaa158 PMC7709214 32945868

[45] Ormazabal V (2018). Association between insulin resistance and the development of cardiovascular disease. Cardiovasc. Diabetol..

[46] Guerra, I. M. S. *et al.* Mitochondrial fatty acid β-oxidation disorders: From disease to lipidomic studies—A critical review. *Int. J. Mol. Sci.* **23** (2022). 10.3390/ijms232213933 PMC9696092 36430419

[47] Schlierf G, Dorow E (1973). Diurnal patterns of triglycerides, free fatty acids, blood sugar, and insulin during carbohydrate-induction in man and their modification by nocturnal suppression of lipolysis. J. Clin. Investig..

[48] Tortosa-Caparrós E, Navas-Carrillo D, Marín F, Orenes-Piñero E (2017). Anti-inflammatory effects of omega 3 and omega 6 polyunsaturated fatty acids in cardiovascular disease and metabolic syndrome. Crit. Rev. Food Sci. Nutr..

[49] Sinha S, Haque M, Lugova H, Kumar S (2023). The effect of omega-3 fatty acids on insulin resistance. Life.

[50] Taouis M (2002). N-3 Polyunsaturated fatty acids prevent the defect of insulin receptor signaling in muscle. Am. J. Physiol. Endocrinol. Metab..

[51] Bamford NJ (2019). Clinical insights: Treatment of laminitis. Equine Vet. J..

[52] Respondek F, Myers K, Smith TL, Wagner A, Geor RJ (2011). Dietary supplementation with short-chain fructo-oligosaccharides improves insulin sensitivity in obese horses. J. Anim. Sci..

[53] McGowan CM, Dugdale AH, Pinchbeck GL, Argo CMG (2013). Dietary restriction in combination with a nutraceutical supplement for the management of equine metabolic syndrome in horses. Vet. J..

[54] Manfredi, J. M., Stapley, E. D., Nadeau, J. A. & Nash, D. Investigation of the effects of a dietary supplement on insulin and adipokine concentrations in equine metabolic syndrome/insulin dysregulation. *J. Equine Vet Sci.* **88** (2020). 10.1016/j.jevs.2020.102930 32303322

[55] Indiveri C (2011). The mitochondrial carnitine/acylcarnitine carrier: Function, structure and physiopathology. Mol. Asp. Med..

[56] Virmani, M. A. & Cirulli, M. The role of L-carnitine in mitochondria, prevention of metabolic inflexibility and disease initiation. *Int. J. Mol. Sci.* **23** (2022). 10.3390/ijms23052717 PMC8910660 35269860

[57] Sangouni AA (2022). The effect of l-carnitine supplementation on insulin resistance, sex hormone-binding globulin and lipid profile in overweight/obese women with polycystic ovary syndrome: A randomized clinical trial. Eur. J. Nutr..

[58] Xu Y (2017). L-carnitine treatment of insulin resistance: A systematic review and meta-analysis. Adv. Clin. Exp. Med..

[59] Kranenburg LC (2014). The effect of long-term oral L-carnitine administration on insulin sensitivity, glucose disposal, plasma concentrations of leptin and acylcarnitines, and urinary acylcarnitine excretion in warmblood horses. Vet Q..

[60] Abdali D, Samson SE, Grover AK (2015). How effective are antioxidant supplements in obesity and diabetes?. Med. Princ. Pract..

[61] Formoso, G. *et al.* Inositol and antioxidant supplementation: Safety and efficacy in pregnancy. *Diabetes Metab. Res. Rev.* **35** (2019). 10.1002/dmrr.3154 PMC6617769 30889626

[62] Gülçin I (2006). Antioxidant and antiradical activities of L-carnitine. Life Sci..

[63] Ribas GS, Vargas CR, Wajner M (2014). L-carnitine supplementation as a potential antioxidant therapy for inherited neurometabolic disorders. Gene.

[64] Patel MS, Korotchkina LG (2001). Regulation of mammalian pyruvate dehydrogenase complex by phosphorylation: Complexity of multiple phosphorylation sites and kinases. Exp. Mol. Med..

[65] Henneke DR, Potter GD, Kreider JL, Yeates BF (1983). Relationship between condition score, physical measurements and body fat percentage in mares. Equine Vet. J..

[66] Carter RA, Geor RJ, Burton Staniar W, Cubitt TA, Harris PA (2009). Apparent adiposity assessed by standardised scoring systems and morphometric measurements in horses and ponies. Vet. J..

[67] Gentry LR (2004). The relationship between body condition score and ultrasonic fat measurements in mares of high versus low body condition. J. Equine Vet. Sci..

[68] Kane, R. A., Fisher, M., Parrett, D. & Lawrence L. M. *Proceedings of the 10th Equine Nutrition and Physiology Symposium, June 11–13, 1987, the Fort Collins Marriott, Colorado State University.* (Equine Nutrition and Physiology Society, 1987).

[69] Treiber KH, Kronfeld DS, Hess TM, Boston RC, Harris PA (2005). Use of proxies and reference quintiles obtained from minimal model analysis for determination of insulin sensitivity and pancreatic beta-cell responsiveness in horses. Am. J. Vet. Res..

[70] Harris WS, Pottala JV, Vasan RS, Larson MG, Robins SJ (2012). Changes in erythrocyte membrane trans and marine fatty acids between 1999 and 2006 in older Americans. J. Nutr..

[71] Metabolomics H-T (2019). Methods and Protocols.

[72] Li Puma LC (2020). Experimental oxygen concentration influences rates of mitochondrial hydrogen peroxide release from cardiac and skeletal muscle preparations. Am. J. Physiol. Regul. Integr. Comp. Physiol..

[73] Pesta D, Gnaiger E (2011). High-resolution respirometry: OXPHOS protocols for human cells and permeabilized fibers from small biopsies of human muscle. Methods Mol Biol (Clifton, NJ).

[74] Chicco AJ (2018). Adaptive remodeling of skeletal muscle energy metabolism in high-altitude hypoxia: lessons from AltitudeOmics. J Biol. Chem..

[75] Dieckmann-Schuppert A, Schnittler H-J (1997). A simple assay for quantification of protein in tissue sections, cell cultures, and cell homogenates, and of protein immobilized on solid surfaces. Cell Tissue Res..

