# Supplementary material for: Adiposity in mares induces insulin dysregulation and mitochondrial dysfunction which can be mitigated by nutritional intervention
Source: Sci Rep. 2024 Jun 18;14:13992. doi: 10.1038/s41598-024-64628-x (PMC11183153; doi:10.1038/s41598-024-64628-x)
Supplement: Supplementary file 1 — Supplementary Figures. [file 41598_2024_64628_MOESM1_ESM.docx]

**Adiposity in mares induces insulin dysregulation and mitochondrial dysfunction which can be mitigated by nutritional intervention**

Kyle Fresa, Giovana D Catandi, Luke Whitcomb, Raul A. Gonzalez-Castro, Adam J Chicco, Elaine M Carnevale^*^

Department of Biomedical Sciences, Colorado State University, Fort Collins, CO 80523, USA.

^*^Corresponding author. Email: [elaine.carnevale@colostate.edu](mailto:elaine.carnevale@colostate.edu)

**Supplementary Figure S1. Morphometric and metabolic classification of mares.** Phenotypic measurements from normal weight (NW), obese (OB) and obese fed the Diet Supplement (ODB) mares performed at 2-week intervals: (**a**) body condition score (BCS), (**b**) percentage of body fat (BF) calculated using a tailhead fat measurement, (**c**) cresty neck score (NS), and (**d**) body weight (BW) in kg. ^ab^ Superscripts indicate differences between groups at p < 0.05 for each week. ^AB^ Superscripts indicate differences between weeks at p < 0.05 within individual groups. Graphs represent mean ± SEM.


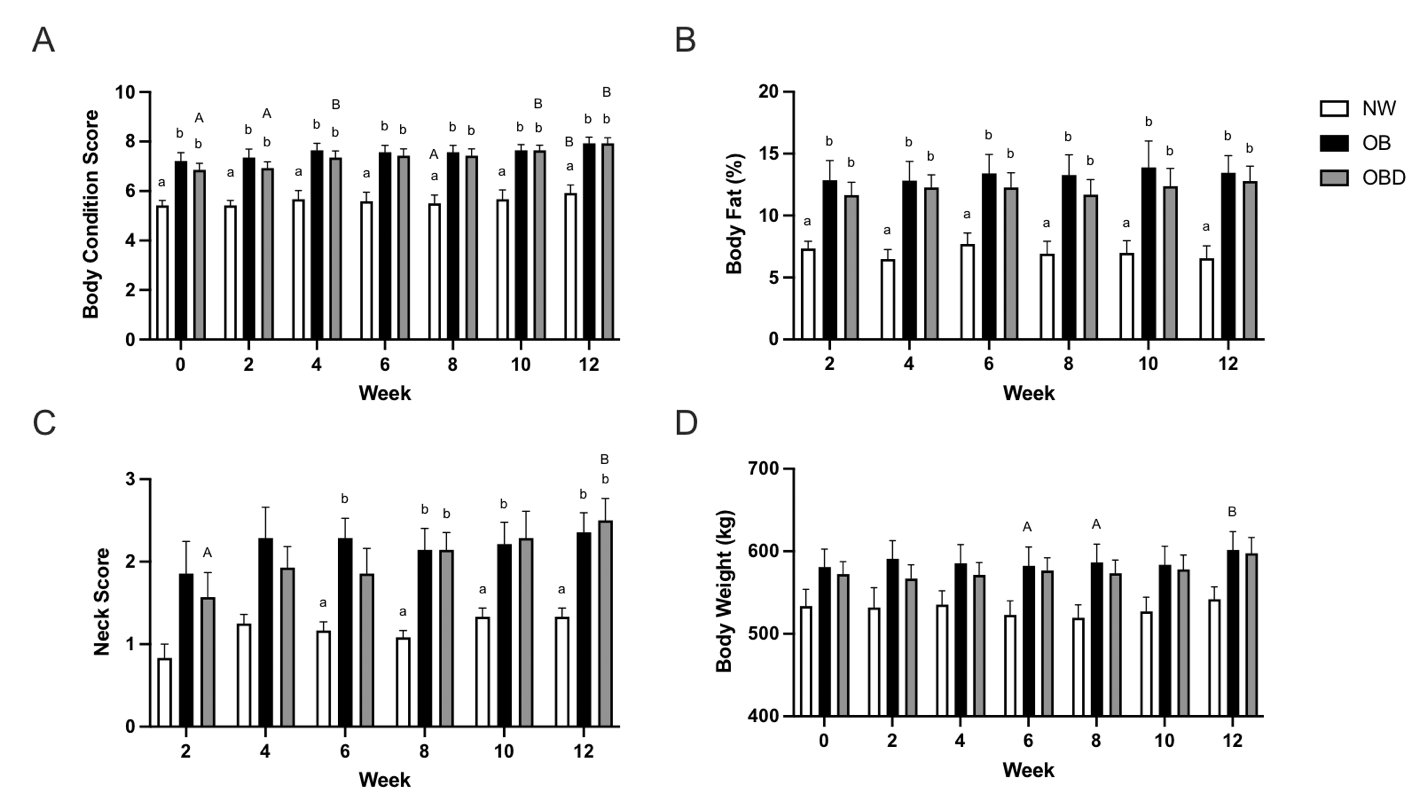


**Supplemental Figure S2:** **Experimental Design.** Three groups of mares were assigned to the study: NW, normal-weight mares; OB, obese mares; OBD, obese mares supplemented with a complex blend of nutrients, including L-carnitine. Mares were maintained on their respective diets for ~12 weeks prior to collection of samples. After all samples were collected, mares in the OB group were maintained on the same diet (hay and grain) with the inclusion of L-carnitine for an additional ~6 weeks prior to another sample collection.

**
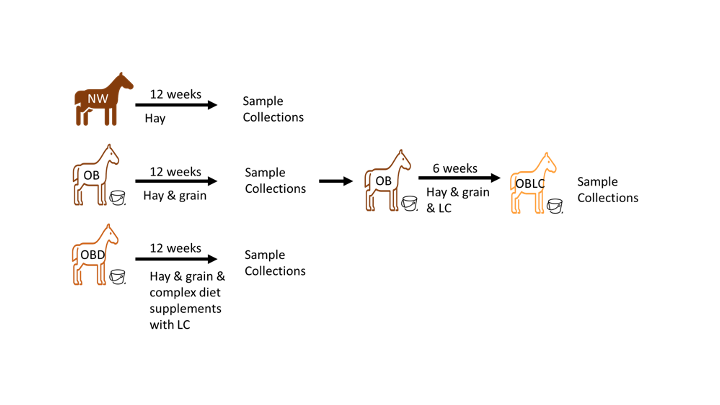
**
