# Supplementary material for: Adiposity in mares induces insulin dysregulation and mitochondrial dysfunction which can be mitigated by nutritional intervention
Source: Sci Rep. 2024 Jun 18;14:13992. doi: 10.1038/s41598-024-64628-x (PMC11183153; doi:10.1038/s41598-024-64628-x)
Supplement: Supplementary file 2 — Supplementary Information 2. [file 41598_2024_64628_MOESM2_ESM.docx]

Uncropped Western blots: SOD1 and SOD2


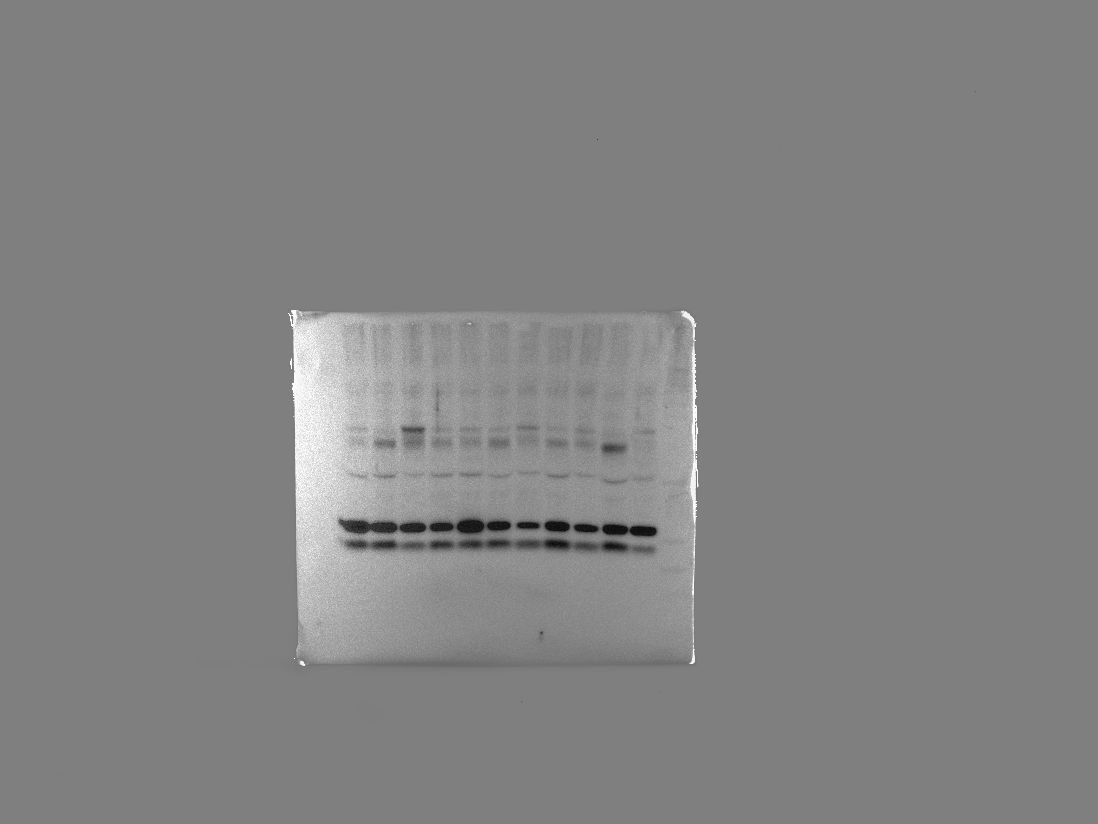


L 1 2 3 4 5 6 7 8 9 10 11

SOD2: 25 kDa

SOD1: 19 kDa


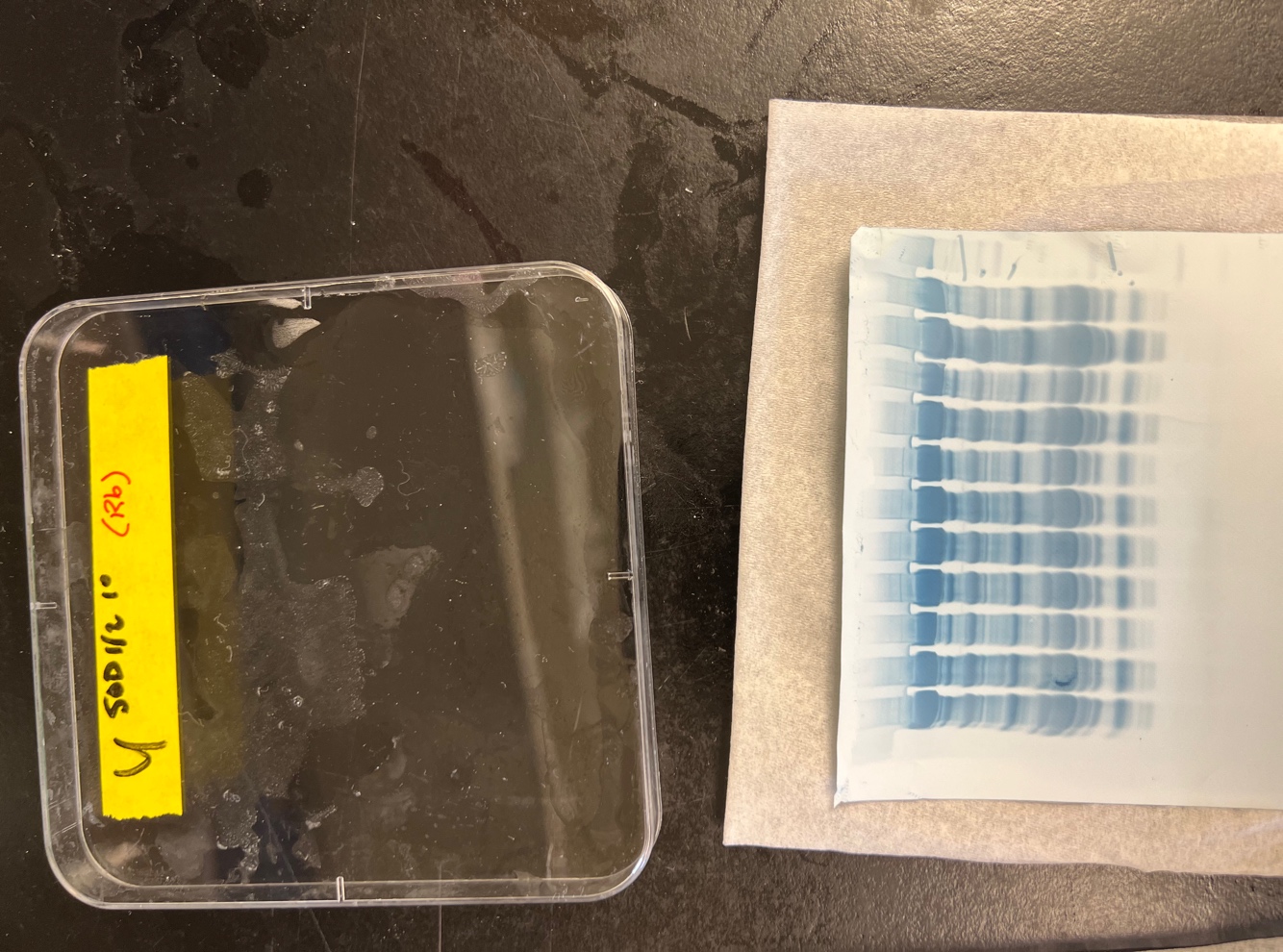


L 1 2 3 4 5 6 7 8 9 10 11

Amido Black total protein stain

L: Ladder

1: NW1

2: OB1

3: OBD1

4: OBLC1

5: NW2

6: OB2

7: OBD2

8: OBLC2

9: NW3

10: OB3

11: NW4


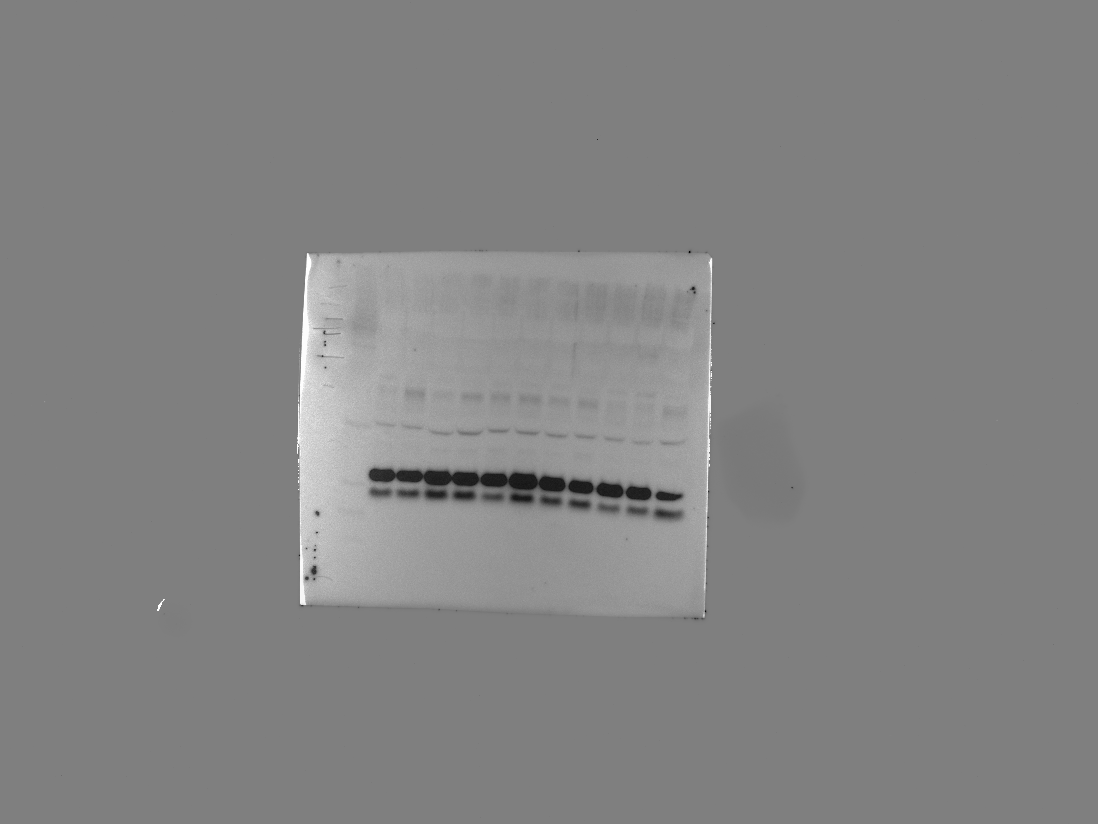


L 1 2 3 4 5 6 7 8 9 10 11

SOD2: 25 kDa

SOD1: 19 kDa


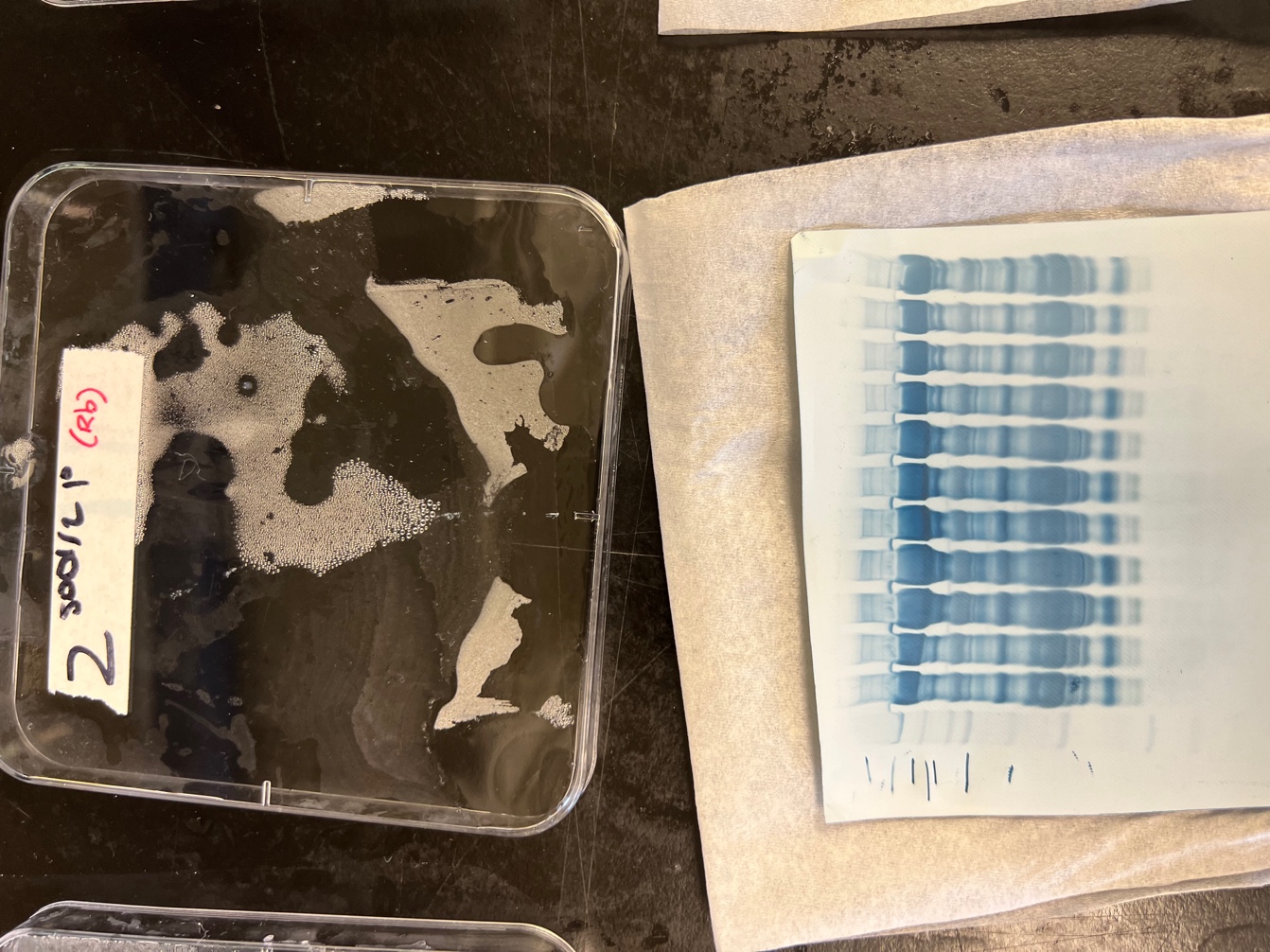


L 1 2 3 4 5 6 7 8 9 10 11

Amido Black total protein stain

L: Ladder

1: NW5

2: OB4

3: OBD3

4: OBLC3

5: NW6

6: OB5

7: OBD4

8: OBLC4

9: NW1

10: OBLC5

11: NW2


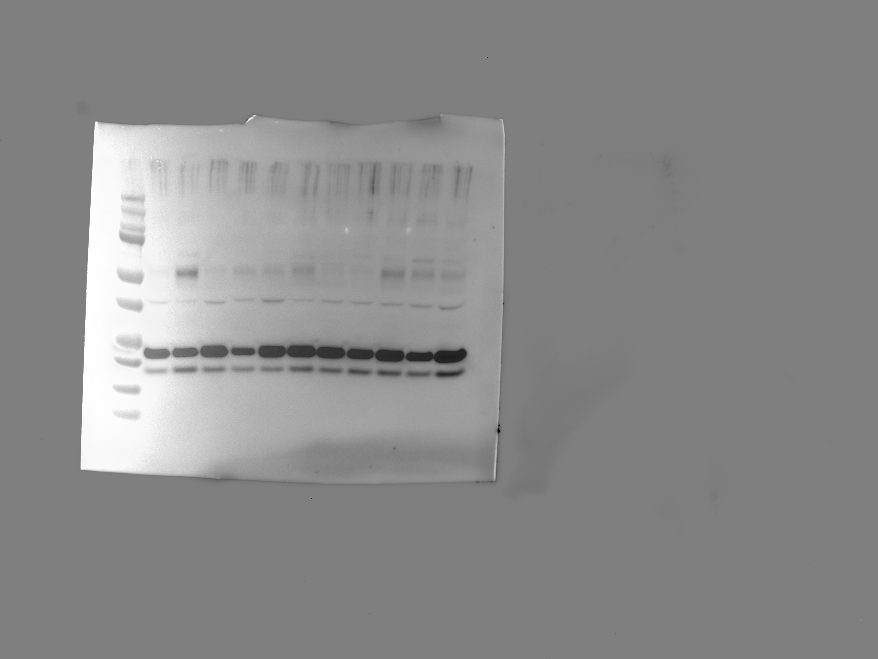


L 1 2 3 4 5 6 7 8 9 10 11

SOD2: 25 kDa

SOD1: 19 kDa


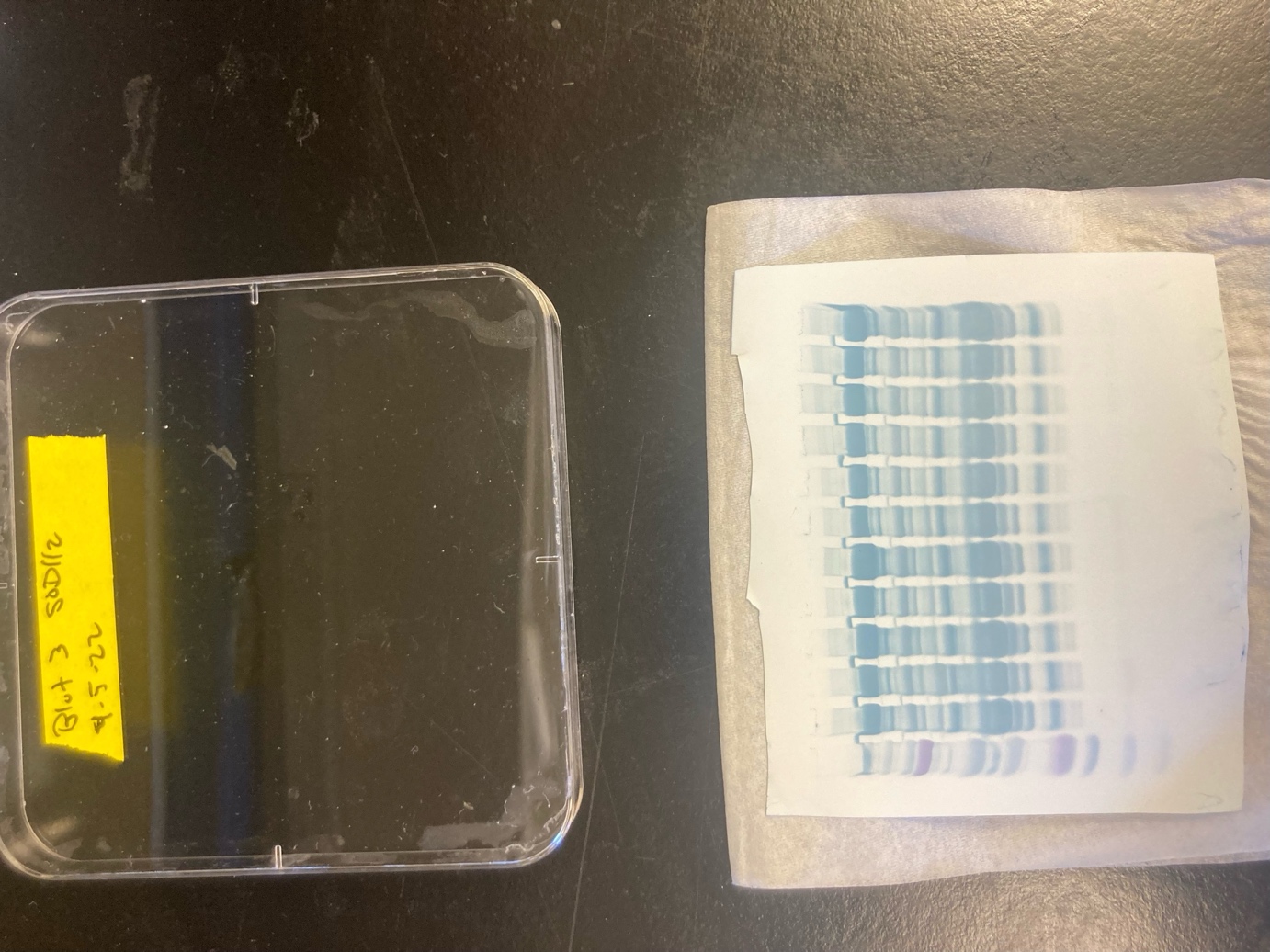


L 1 2 3 4 5 6 7 8 9 10 11

Amido Black total protein stain

L: Ladder

1: NW5

2: OB6

3: OBD5

4: OBLC6

5: NW6

6: OB7

7: OBD6

8: OBLC7

9: NW3

10: OBD7

11: NW6

Uncropped Western blots: pIRS1/IRS1


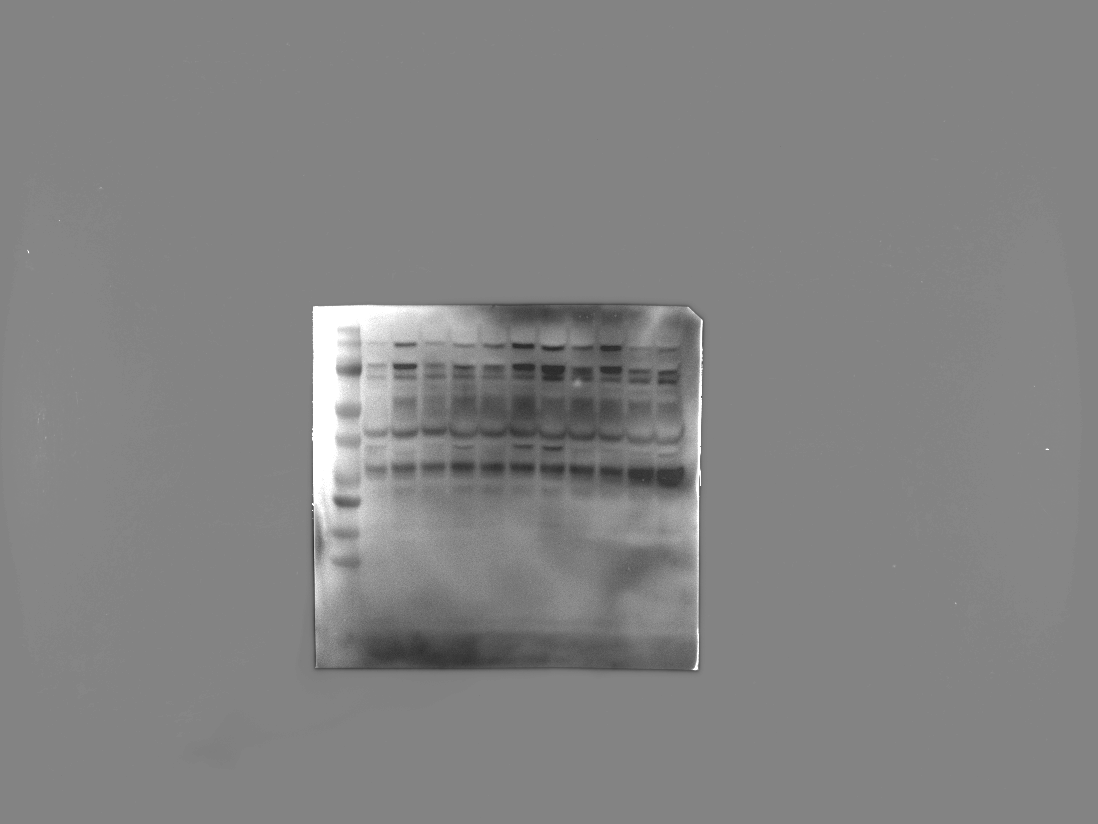


L 1 2 3 4 5 6 7 8 9 10 11

pIRS1: 175 kDa


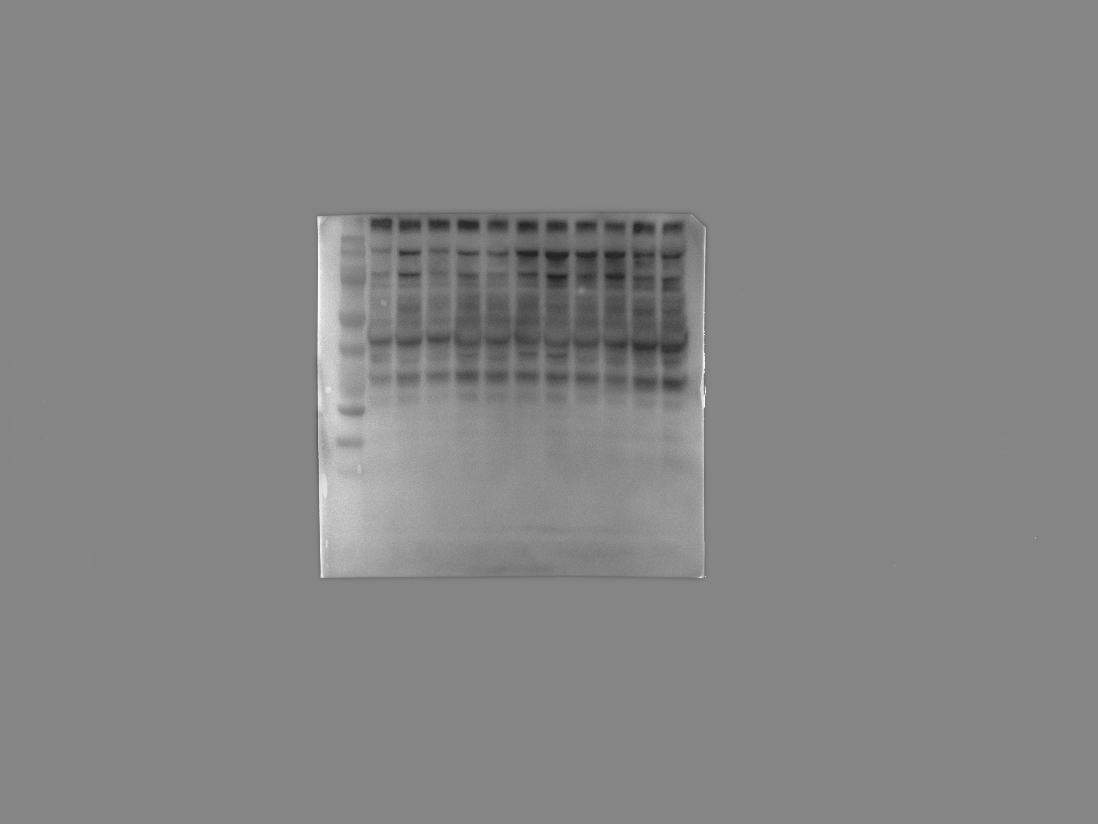
 L 1 2 3 4 5 6 7 8 9 10 11

IRS1: 175 kDa

L: Ladder

1: NW1

2: OB1

3: OBD1

4: OBLC1

5: NW2

6: OB2

7: OBD2

8: OBLC2

9: NW3

10: OB3

11: NW4


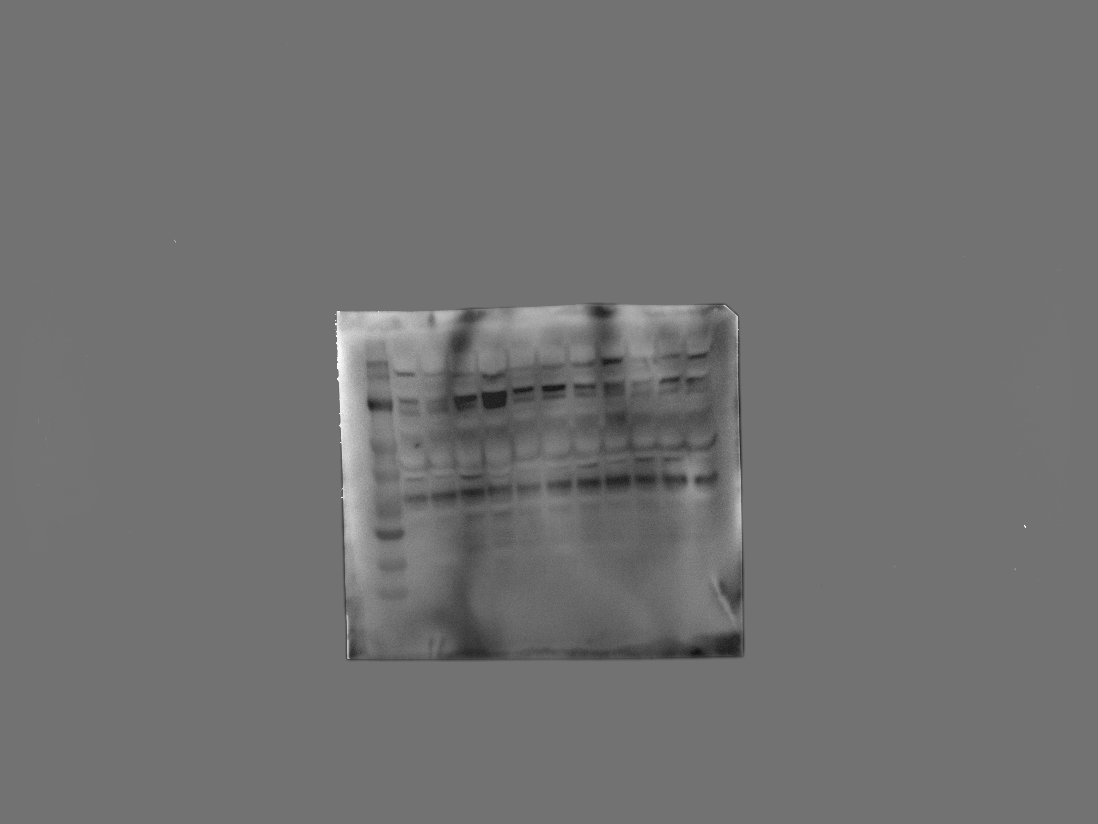


L 1 2 3 4 5 6 7 8 9 10 11

pIRS1: 175 kDa


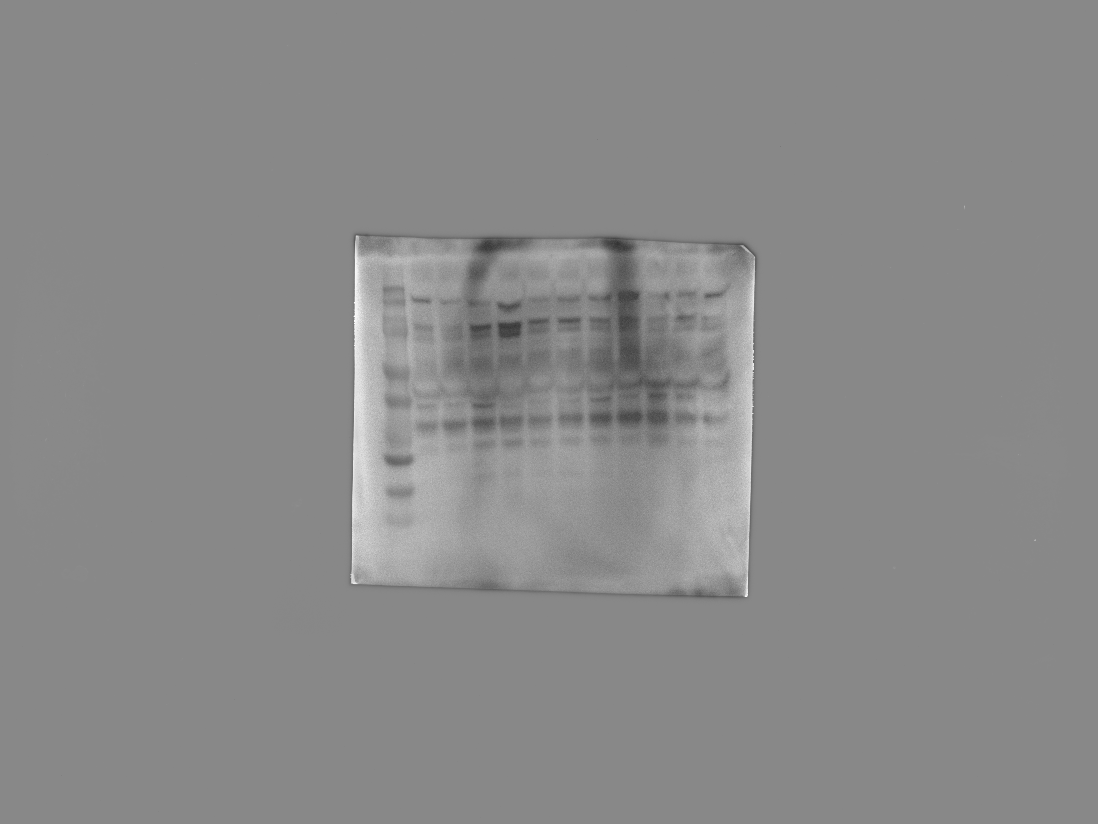


L 1 2 3 4 5 6 7 8 9 10 11

IRS1: 175 kDa

L: Ladder

1: NW5

2: OB4

3: OBD3

4: OBLC3

5: NW6

6: OB5

7: OBD4

8: OBLC4

9: NW1

10: OBLC5

11: NW2


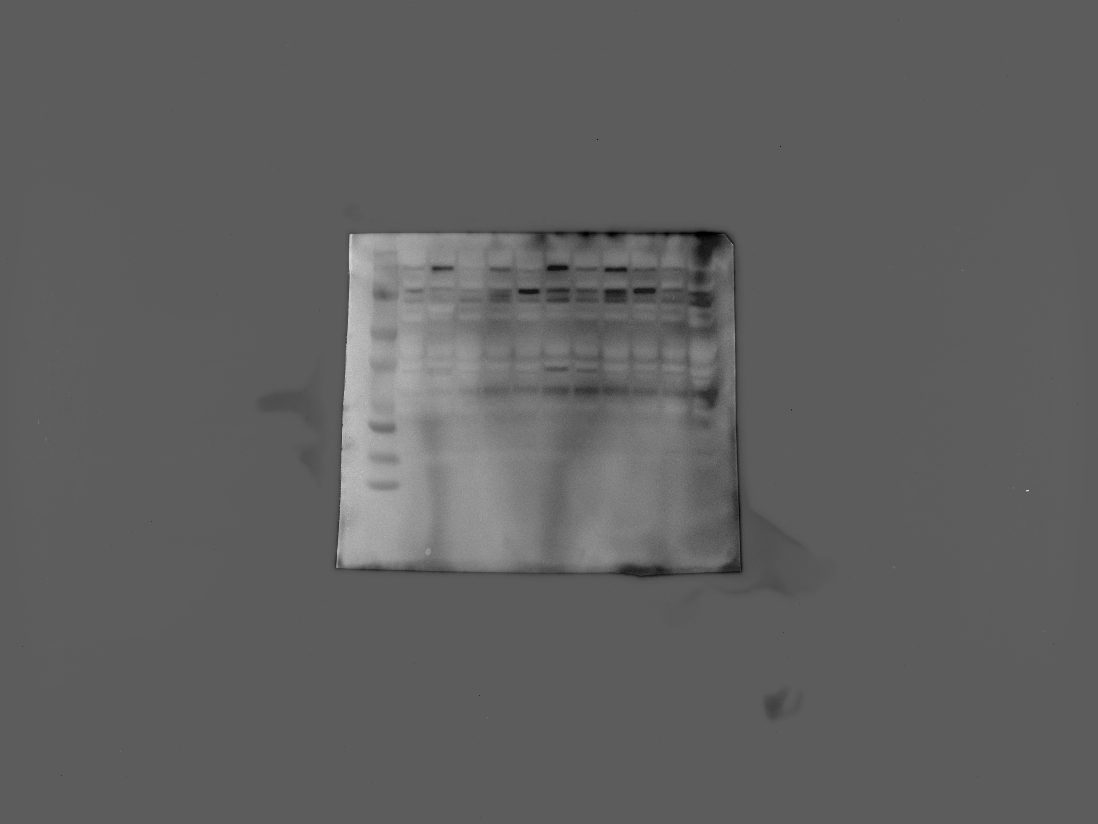


L 1 2 3 4 5 6 7 8 9 10 11

pIRS1: 175 kDa

L 1 2 3 4 5 6 7 8 9 10 11


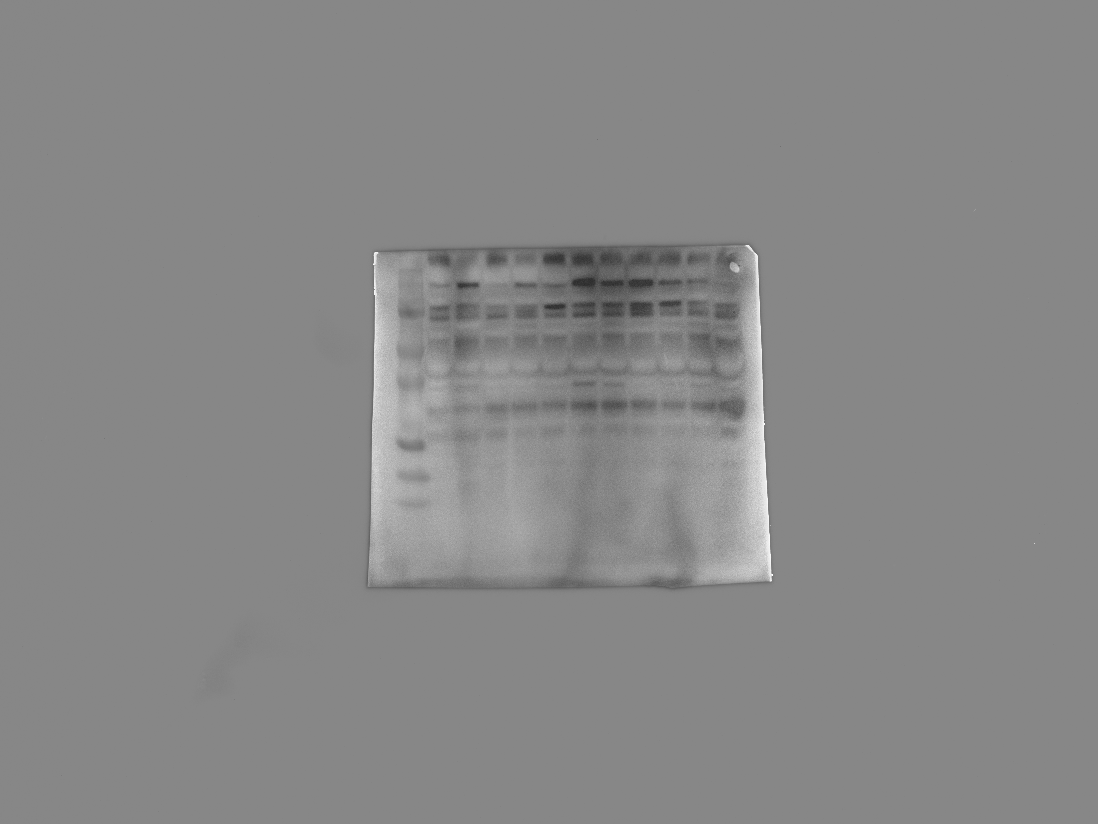


IRS1: 175 kDa

L: Ladder

1: NW5

2: OB6

3: OBD5

4: OBLC6

5: NW6

6: OB7

7: OBD6

8: OBLC7

9: NW3

10: OBD7

11: NW6

Uncropped Western blots: OXPHOS


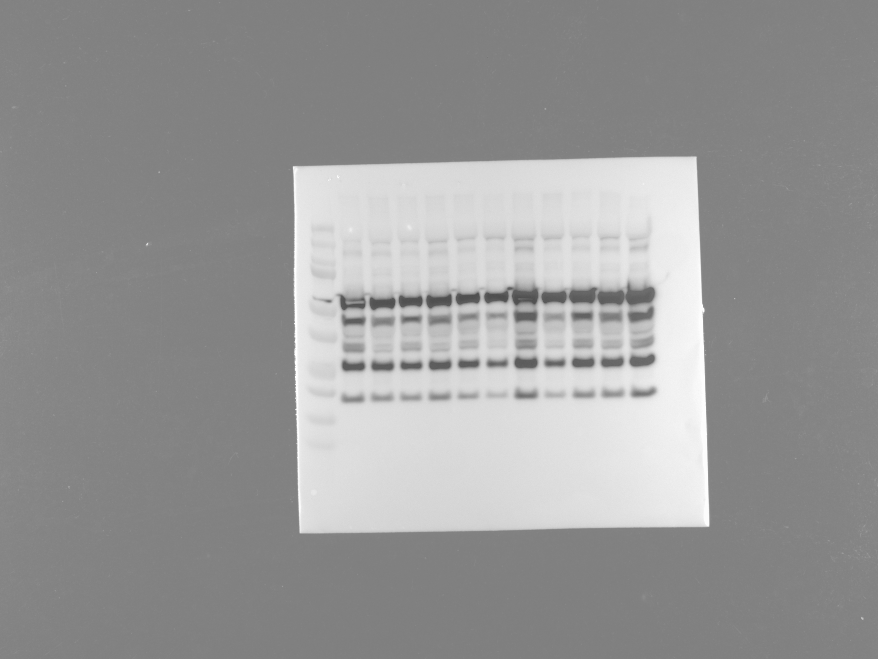


L 1 2 3 4 5 6 7 8 9 10 11

CV: 58 kDa

CIII: 45 kDa

CIV: 35 kDa

CII: 30 kDa

CI: 18 kDa


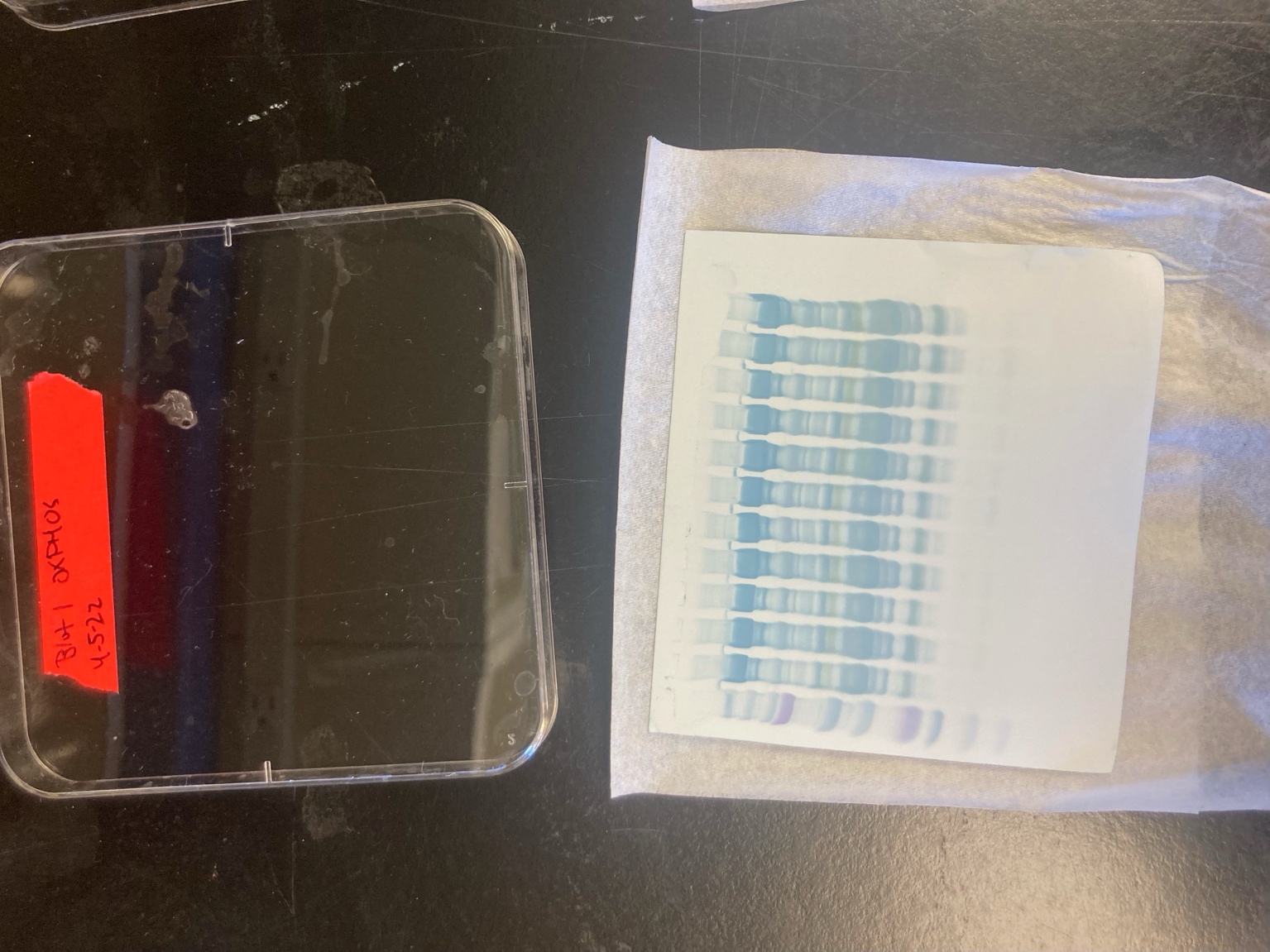


L 1 2 3 4 5 6 7 8 9 10 11

Amido Black total protein stain

L: Ladder

1: NW1

2: OB1

3: OBD1

4: OBLC1

5: NW2

6: OB2

7: OBD2

8: OBLC2

9: NW3

10: OB3

11: NW4


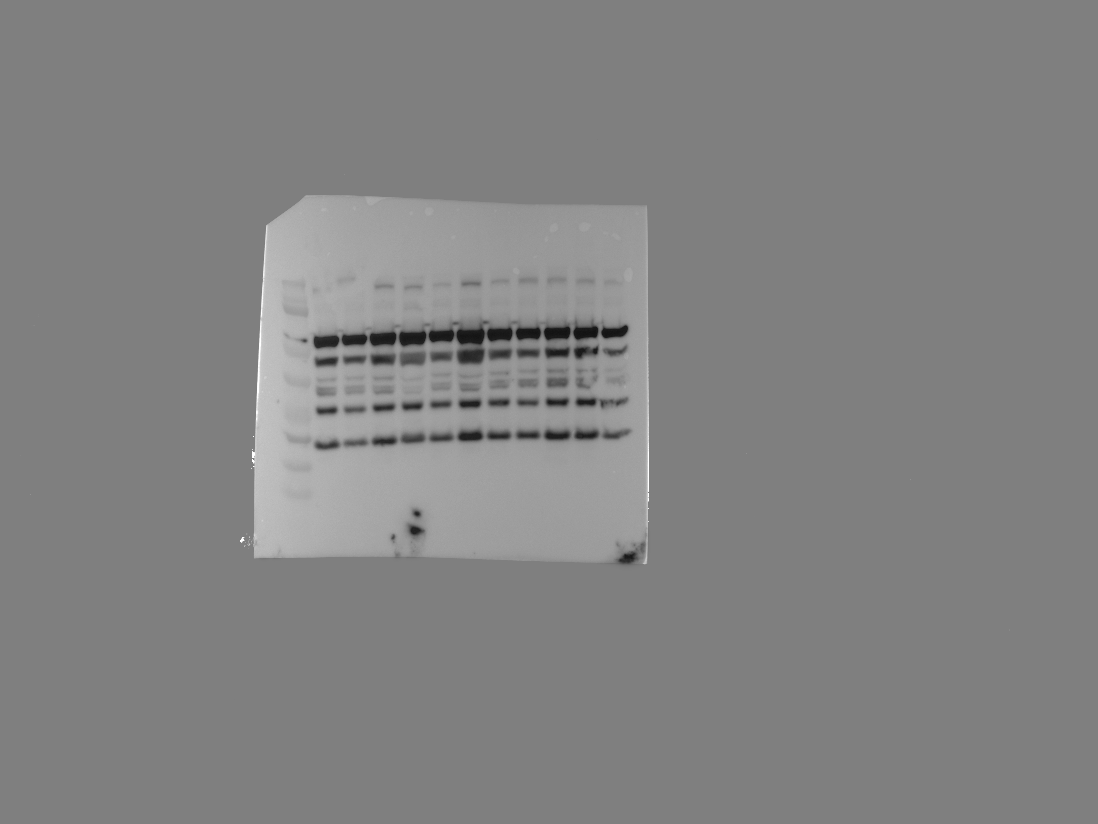


L 1 2 3 4 5 6 7 8 9 10 11

CV: 58 kDa

CIII: 45 kDa

CIV: 35 kDa

CII: 30 kDa

CI: 18 kDa


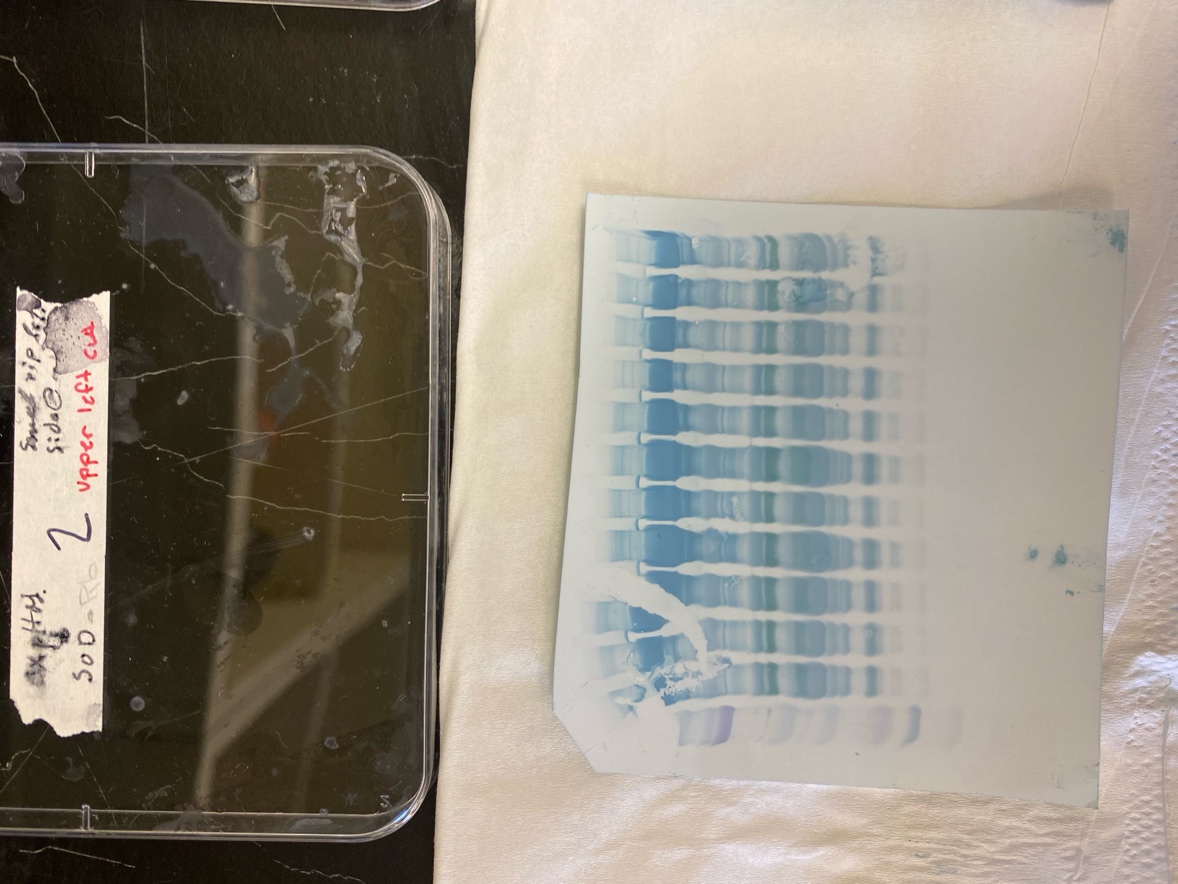


L 1 2 3 4 5 6 7 8 9 10 11

Amido Black total protein stain

L: Ladder

1: NW5

2: OB4

3: OBD3

4: OBLC3

5: NW6

6: OB5

7: OBD4

8: OBLC4

9: NW1

10: OBLC5

11: NW2


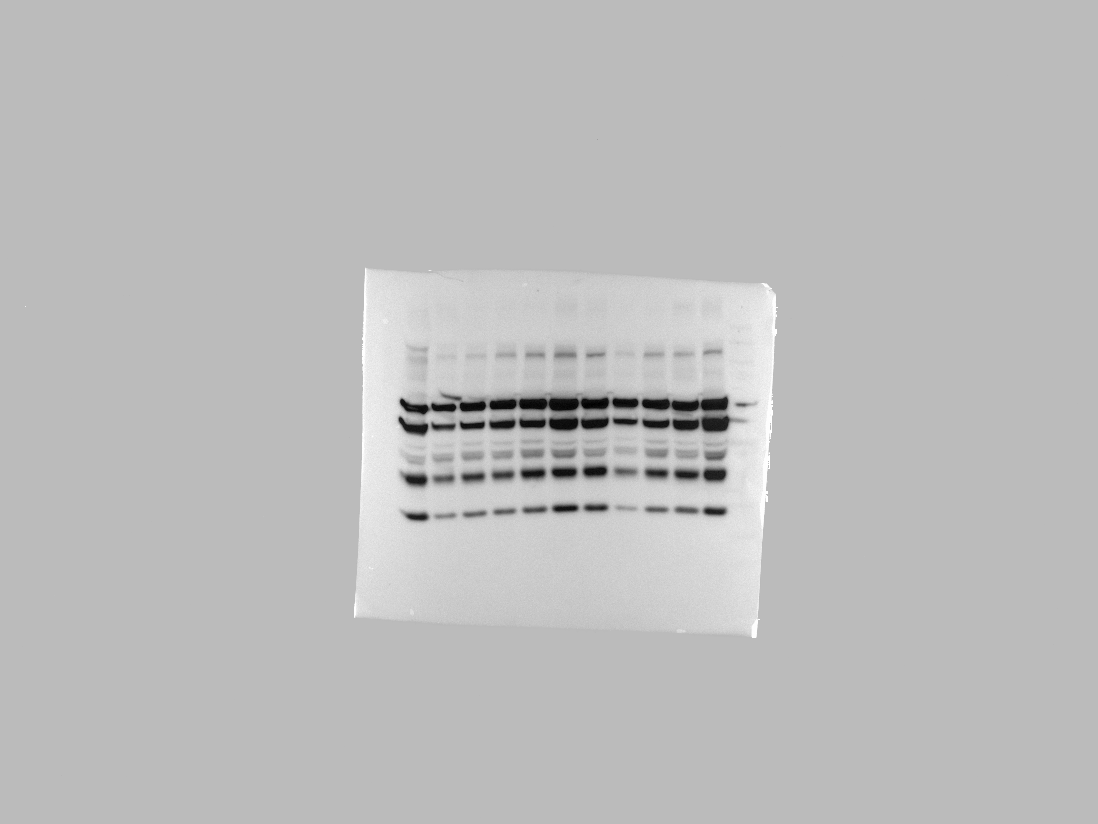


L 1 2 3 4 5 6 7 8 9 10 11

CV: 58 kDa

CIII: 45 kDa

CIV: 35 kDa

CII: 30 kDa

CI: 18 kDa


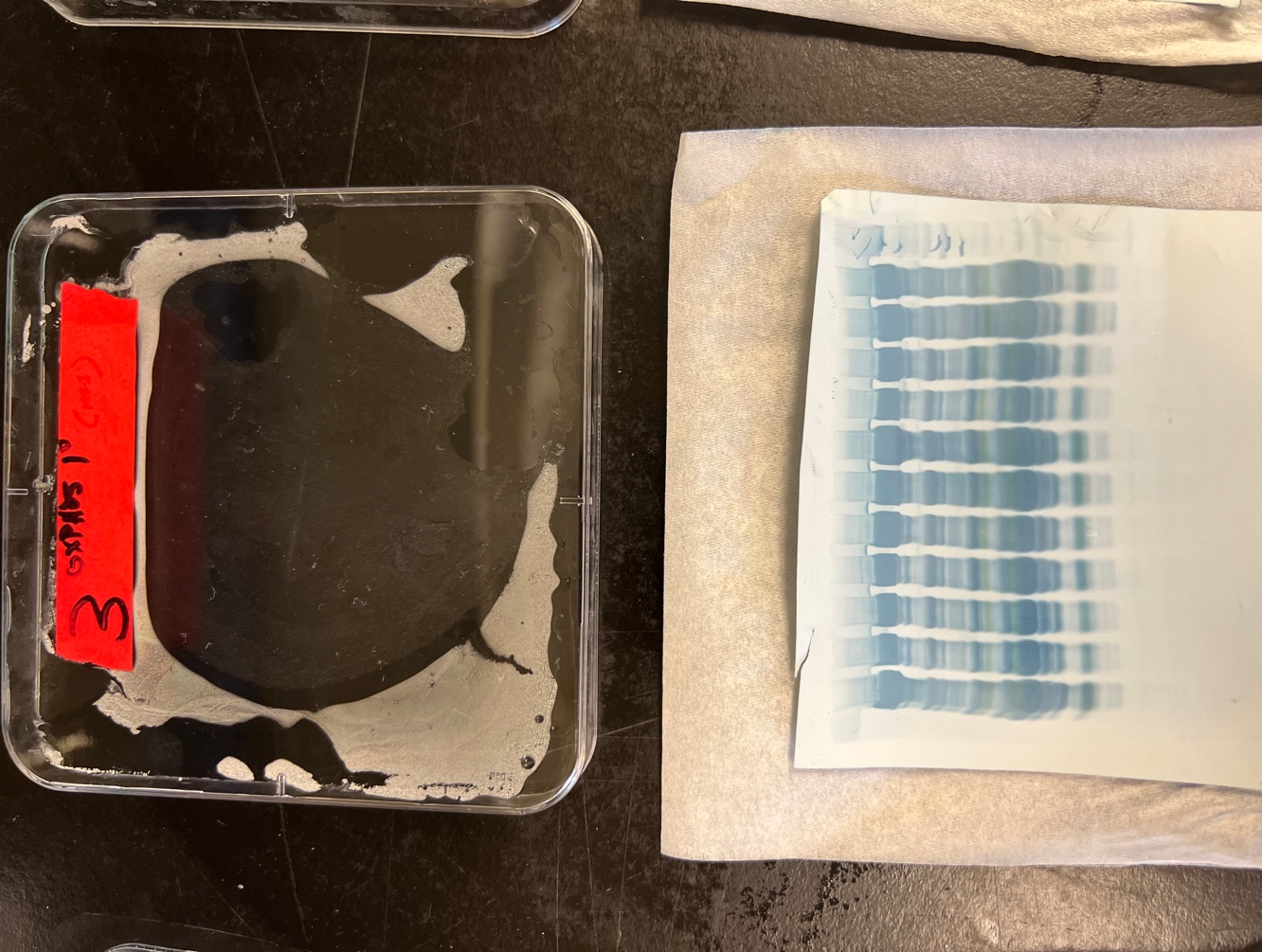


L 1 2 3 4 5 6 7 8 9 10 11

Amido Black total protein stain

L: Ladder

1: NW5

2: OB6

3: OBD5

4: OBLC6

5: NW6

6: OB7

7: OBD6

8: OBLC7

9: NW3

10: OBD7

11: NW6

Uncropped Western blots: VLCAD


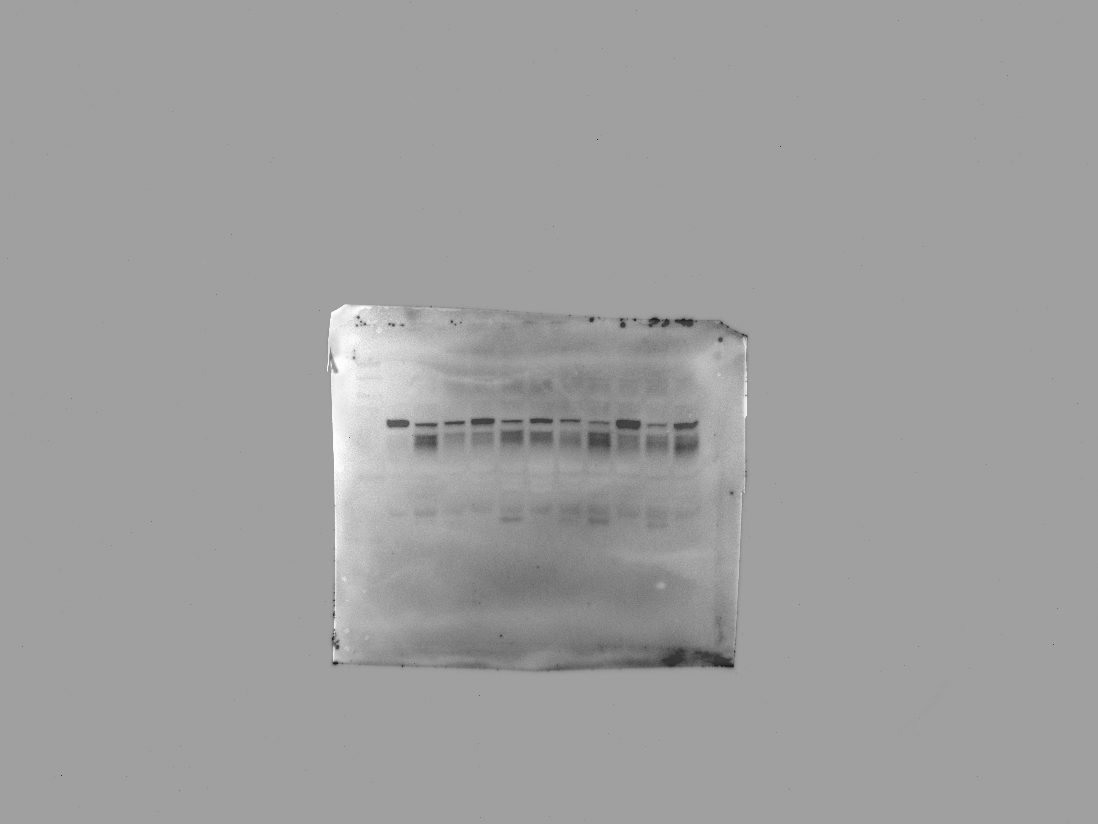


L 1 2 3 4 5 6 7 8 9 10 11

VLCAD: 66 kDa


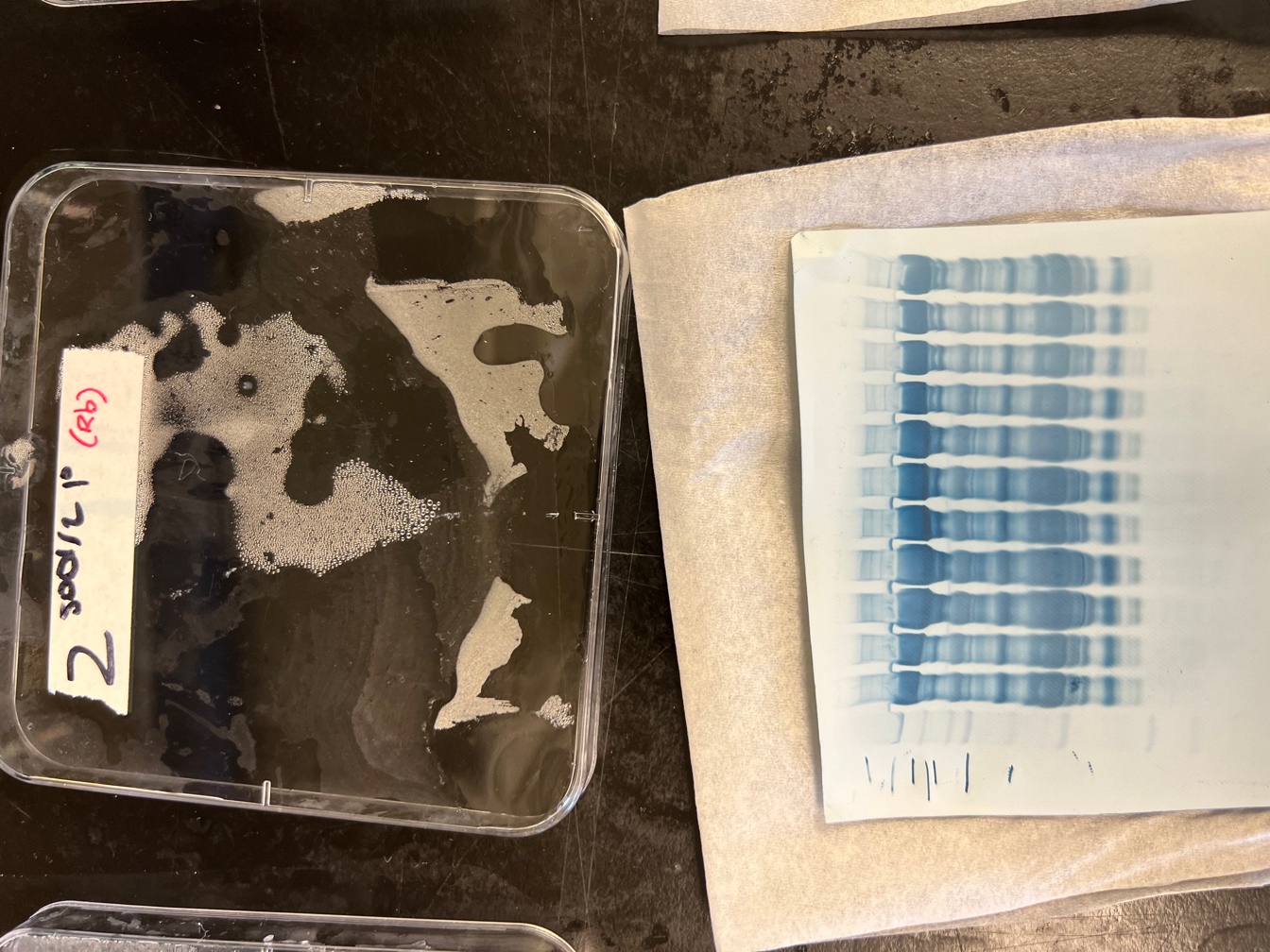


L 1 2 3 4 5 6 7 8 9 10 11

Amido Black total protein stain

L: Ladder

1: NW1

2: OB1

3: OBD1

4: OBLC1

5: NW2

6: OB2

7: OBD2

8: OBLC2

9: NW3

10: OB3

11: NW4


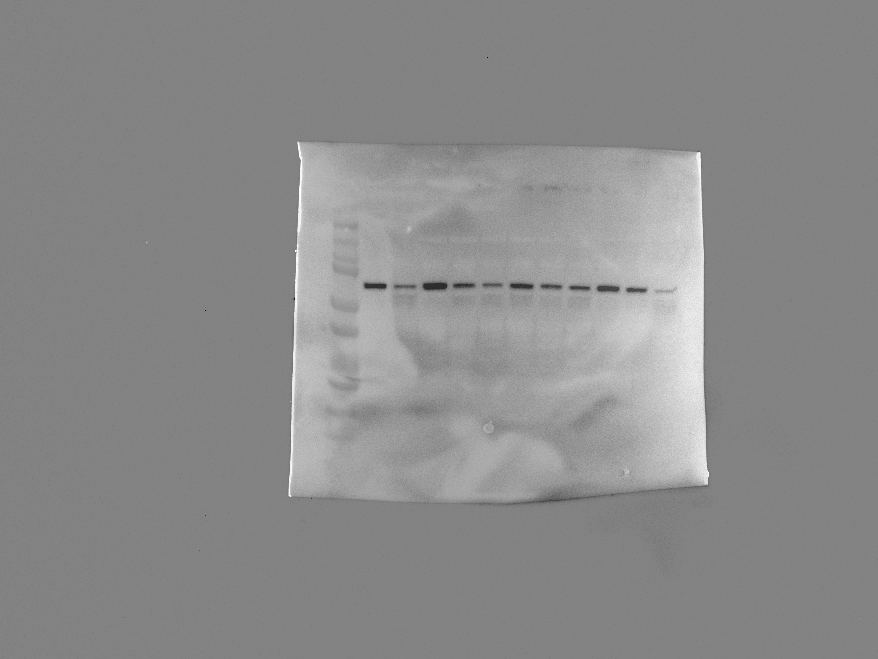


L 1 2 3 4 5 6 7 8 9 10 11

VLCAD: 66 kDa


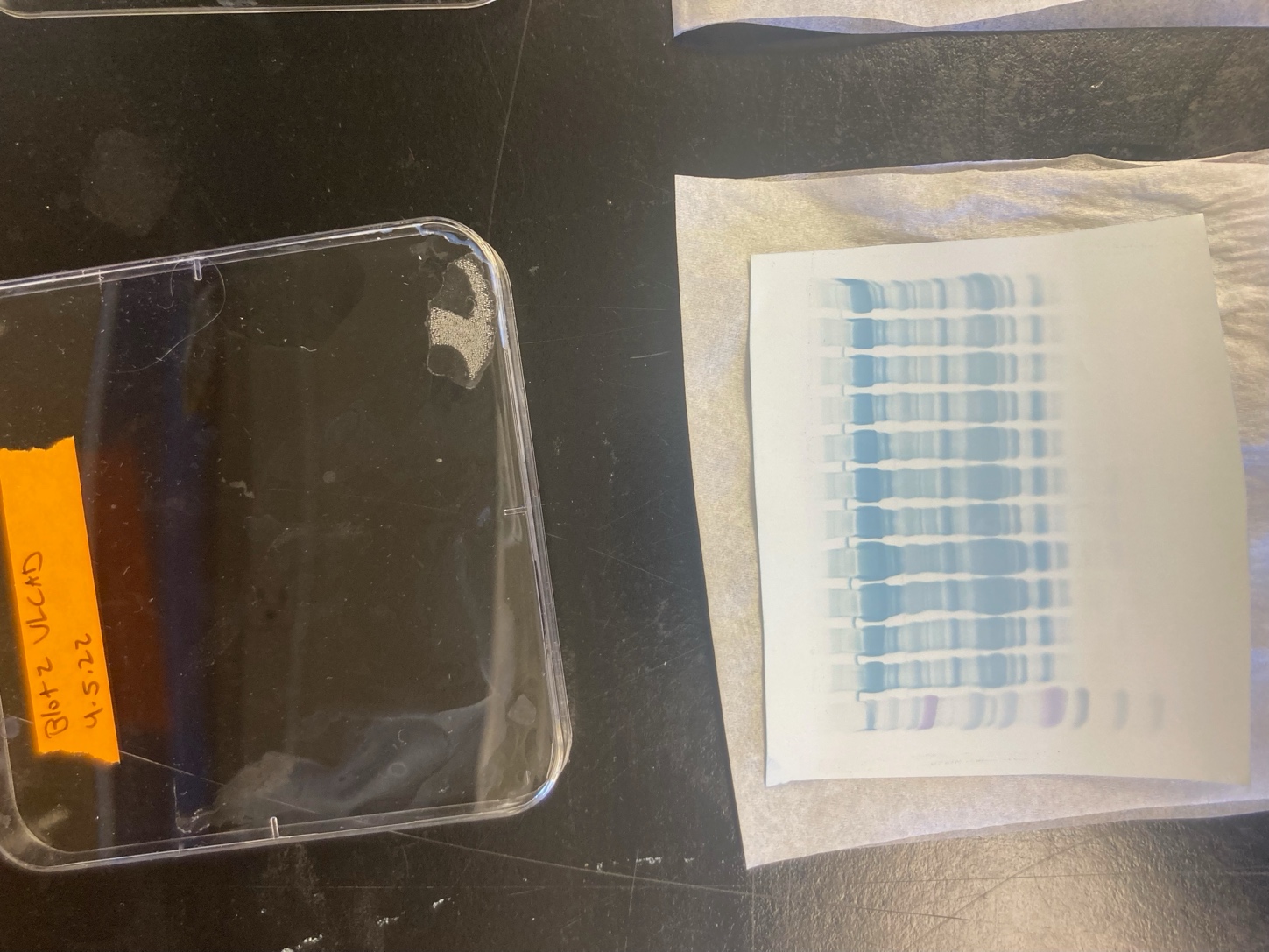


L 1 2 3 4 5 6 7 8 9 10 11

Amido Black total protein stain

L: Ladder

1: NW5

2: OB4

3: OBD3

4: OBLC3

5: NW6

6: OB5

7: OBD4

8: OBLC4

9: NW1

10: OBLC5

11: NW2


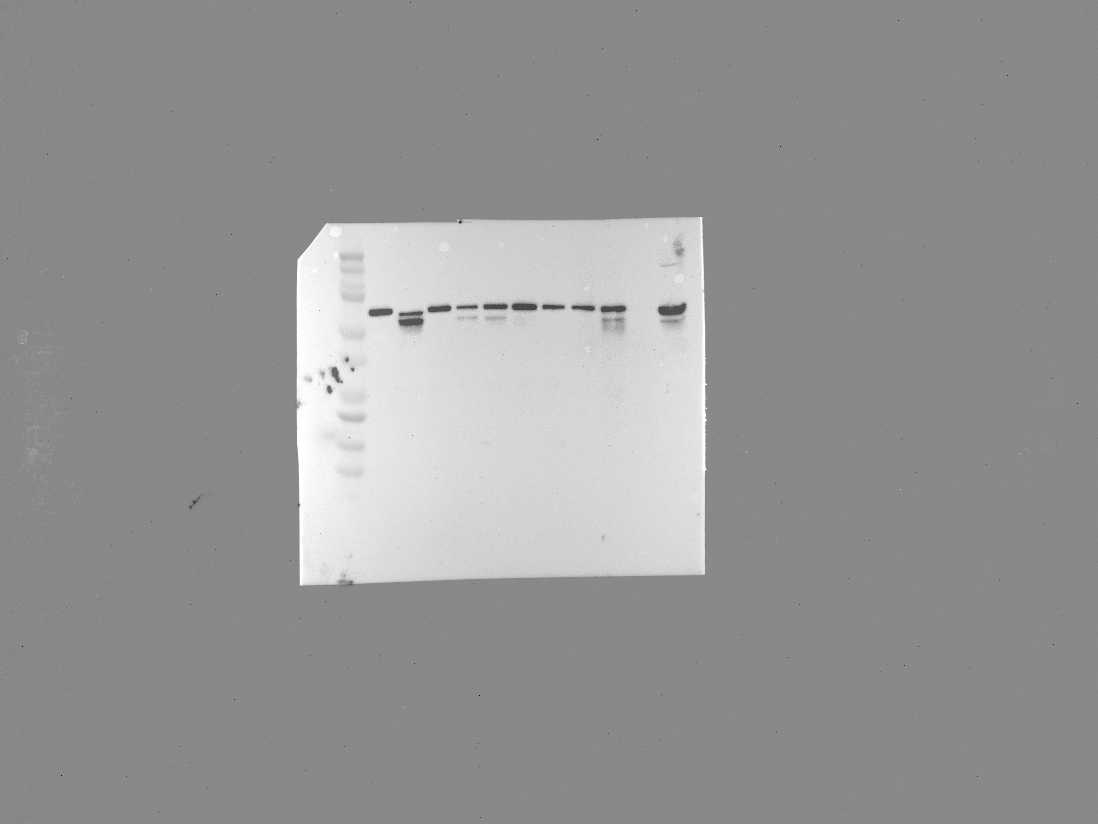


L 1 2 3 4 5 6 7 8 9 10 11

VLCAD: 66 kDa


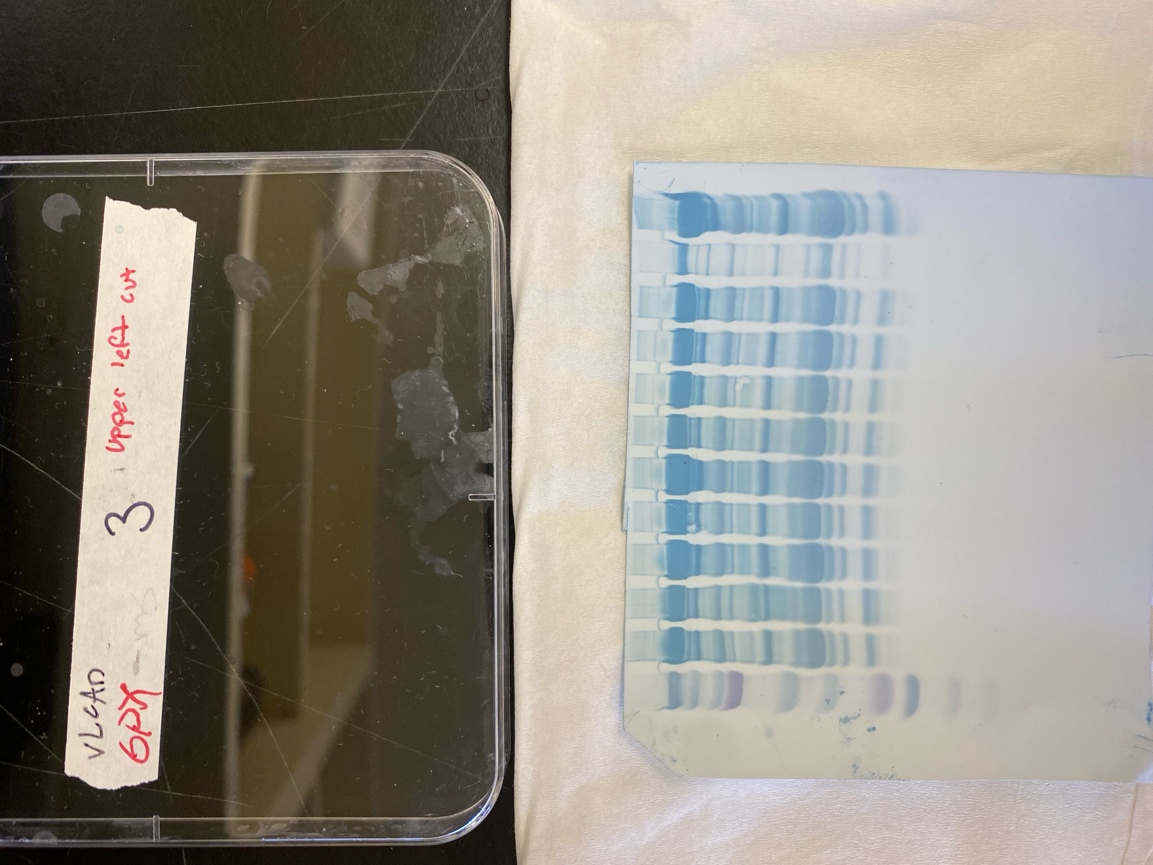


L 1 2 3 4 5 6 7 8 9 10 11

Amido Black total protein stain

L: Ladder

1: NW5

2: OB6

3: OBD5

4: OBLC6

5: NW6

6: OB7

7: OBD6

8: OBLC7

9: NW3

10: OBD7

11: NW6

Uncropped Western blots: pPDH/PDH


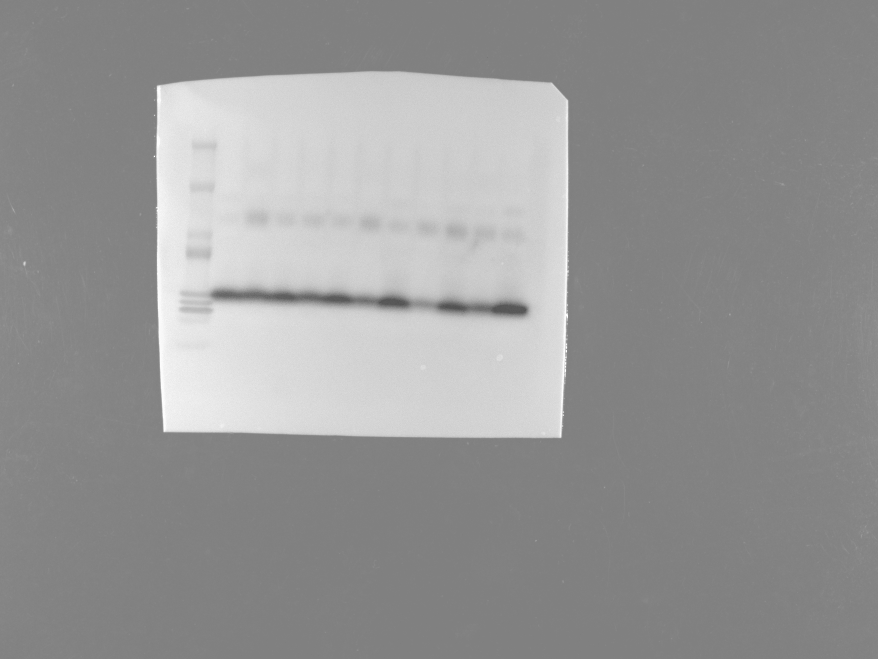


L 1 2 3 4 5 6 7 8 9 10 11

pPDH: 43 kDa


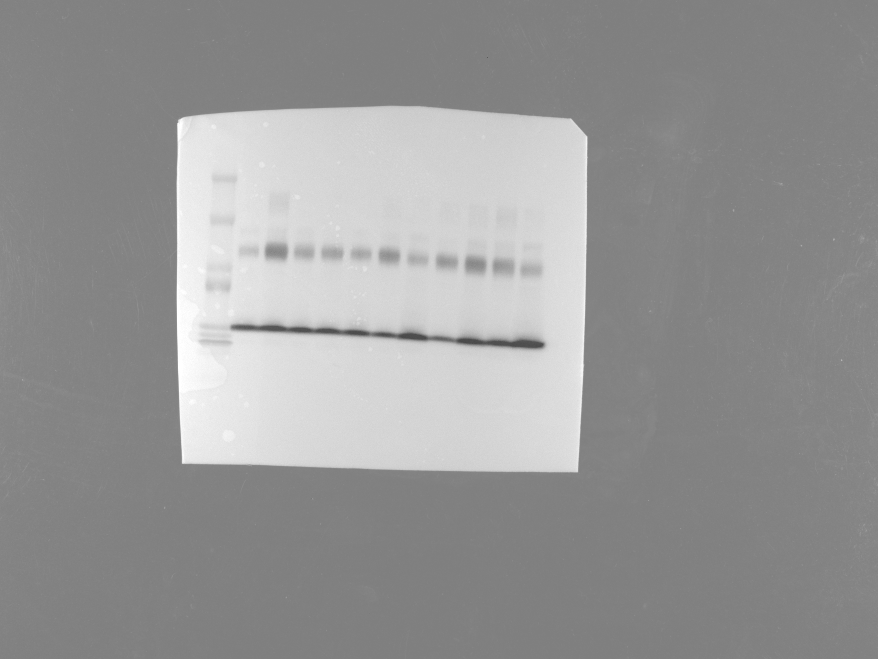


L 1 2 3 4 5 6 7 8 9 10 11

PDH: 39 kDa

L: Ladder

1: NW1

2: OB1

3: OBD1

4: OBLC1

5: NW2

6: OB2

7: OBD2

8: OBLC2

9: NW3

10: OB3

11: NW4


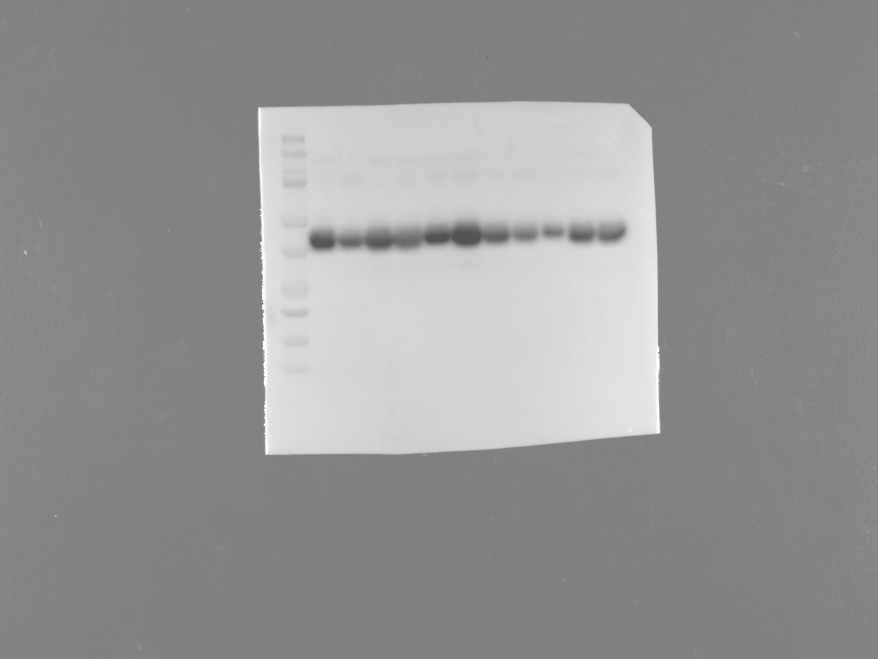


L 1 2 3 4 5 6 7 8 9 10 11

pPDH: 43 kDa


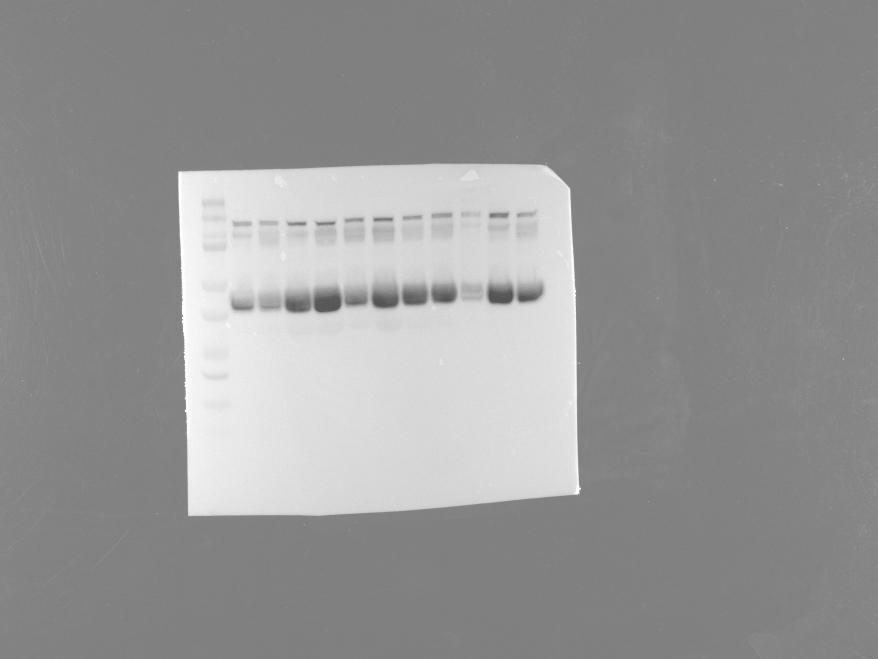


L 1 2 3 4 5 6 7 8 9 10 11

PDH: 39 kDa

L: Ladder

1: NW5

2: OB4

3: OBD3

4: OBLC3

5: NW6

6: OB5

7: OBD4

8: OBLC4

9: NW1

10: OBLC5

11: NW2


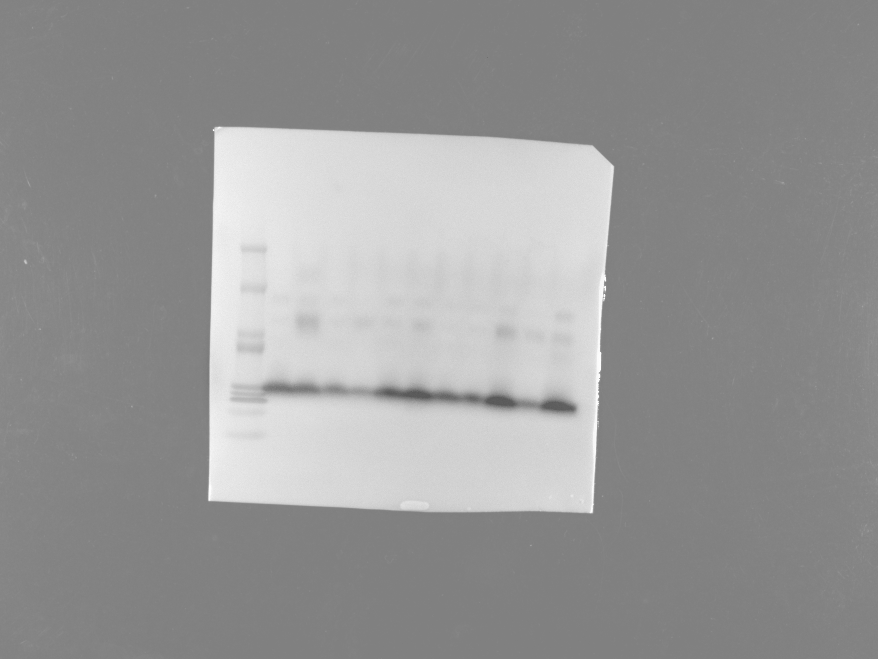


L 1 2 3 4 5 6 7 8 9 10 11

pPDH: 43 kDa


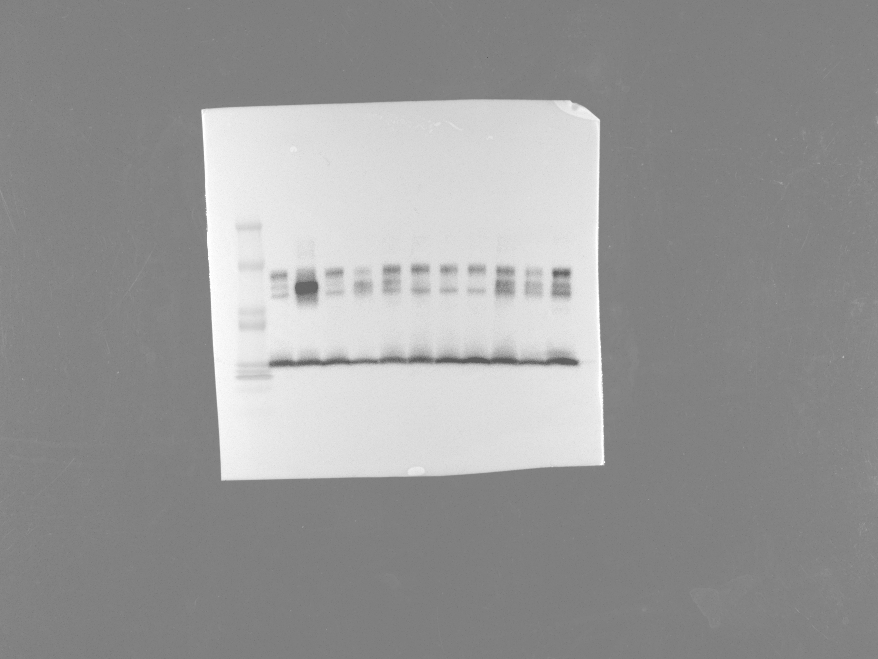


L 1 2 3 4 5 6 7 8 9 10 11

PDH: 39 kDa

L: Ladder

1: NW5

2: OB6

3: OBD5

4: OBLC6

5: NW6

6: OB7

7: OBD6

8: OBLC7

9: NW3

10: OBD7

11: NW6
